# Behavioral characteristics as potential biomarkers of the development and phenotype of epilepsy in a rat model of temporal lobe epilepsy

**DOI:** 10.1038/s41598-021-88088-9

**Published:** 2021-04-21

**Authors:** Karolina Nizinska, Kinga Szydlowska, Avgoustinos Vouros, Anna Kiryk, Aleksandra Stepniak, Eleni Vasilaki, Katarzyna Lukasiuk

**Affiliations:** 1 Laboratory of Epileptogenesis, Nencki Institute of Experimental Biology, Warsaw, Poland; 2 Department of Computer Science, Faculty of Engineering, University of Sheffield, Sheffield, Great Britain; 3 Laboratory of Animal Models, Nencki Institute of Experimental Biology, Warsaw, Poland

**Keywords:** Learning and memory, Epilepsy

## Abstract

The present study performed a detailed analysis of behavior in a rat model of epilepsy using both established and novel methodologies to identify behavioral impairments that may differentiate between animals with a short versus long latency to spontaneous seizures and animals with a low versus high number of seizures. Temporal lobe epilepsy was induced by electrical stimulation of the amygdala. Rats were stimulated for 25 min with 100-ms trains of 1-ms biphasic square-wave pluses that were delivered every 0.5 s. Electroencephalographic recordings were performed to classify rats into groups with a short latency (< 20 days, *n* = 7) and long latency (> 20 days, *n* = 8) to the first spontaneous seizure and into groups with a low number of seizures (62 ± 64.5, *n* = 8) and high number of seizures (456 ± 185, *n* = 7). To examine behavioral impairments, we applied the following behavioral tests during early and late stages of epilepsy: behavioral hyperexcitability, open field, novel object exploration, elevated plus maze, and Morris water maze. No differences in stress levels (e.g., touch response in the behavioral hyperexcitability test), activity (e.g., number of entries into the open arms of the elevated plus maze), or learning (e.g., latency to find the platform in the Morris water maze test during training days) were observed between animals with a short versus long latency to develop spontaneous seizures or between animals with a low versus high number of seizures. However, we found a higher motor activity measured by higher number of entries into the closed arms of the elevated plus maze at week 26 post-stimulation in animals with a high number of seizures compared with animals with a low number of seizures. The analysis of the Morris water maze data categorized the strategies that the animals used to locate the platform showing that the intensity of epilepsy and duration of epileptogenesis influenced swimming strategies. These findings indicate that behavioral impairments were relatively mild in the present model, but some learning strategies may be useful biomarkers in preclinical studies.

## Introduction

Epilepsy is one of the most common brain disorders, affecting 1% of the world’s population^1^. Current data from the World Health Organization indicate that 50 million people suffer from epilepsy (https://www.who.int/health-topics/epilepsy; accessed December 4, 2020). Each year, approximately 2.4 million new cases of epilepsy are diagnosed^2,3^. In 30% of cases, epilepsy develops as a result of brain insult, most commonly stroke, mechanical trauma, and status epilepticus^4^. The initial insult is followed by a latency period (referred to as epileptogenesis) to the first spontaneous seizure. Epileptogenesis is a process by which several neurobiological events occur, including molecular, cellular, and functional changes, following brain damage^5,6^.

In preclinical and clinical studies of human epilepsy and animal models of epilepsy, electroencephalography (EEG) is used for seizure diagnosis. Electroencephalography measures the frequency and intensity of recurrent spontaneous seizures and can predict upcoming seizure events^7^. Electroencephalography in human clinical studies is a minimal criterion for an epilepsy diagnosis^7^. Electroencephalography is also frequently used in animal studies to characterize the development of epilepsy and disease phenotypes. However, the generation of reliable data often requires manual analysis or at least the manual validation of EEG recordings, which can be time-consuming and laborious. This makes EEG an inconvenient tool for rapid drug screening or long-term observations of animal cohorts, thus justifying the search for new, noninvasive biomarkers.

In the present study, we searched for noninvasive biomarkers of epilepsy that might be useful for the diagnosis of epilepsy and prediction of epileptogenesis. In animal models of epilepsy, behavioral and cognitive impairments are frequently observed. We sought to identify noninvasive biomarkers by evaluating whether behavioral impairments depend on the latency to the first spontaneous seizure and intensity of epilepsy, measured as the total number of seizures.

## Materials and methods

### Animal surgery and status epilepticus induction

**Figure 1:**
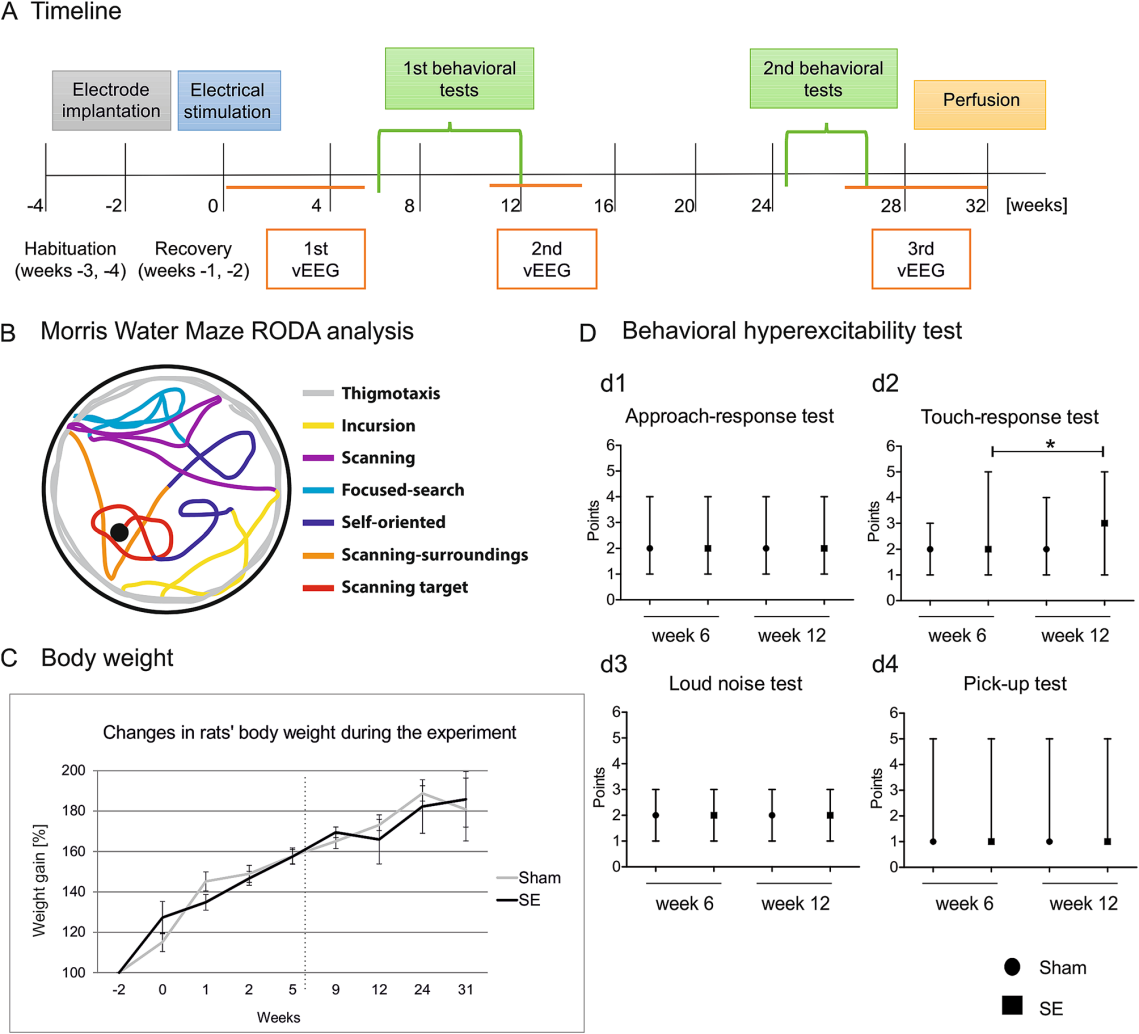
Experimental design, swimming strategies in the Morris water maze, and the animals’ wellbeing during the experiment between sham and stimulated rats. **(A)** Experimental design. Timeline of electrode implantation 2 weeks before electrical stimulation (0 week-start), video EEG (vEEG) monitoring, the battery of behavioral tests, and perfusion (32 week-end). **(B)** Schematic illustration of swimming strategies in the Morris water maze using Rodent Data Analysis (RODA) software. **(C)** Body weight in sham (*n* = 12) and stimulated animals (SE, *n* = 15) during 31 weeks after electrical stimulation. No differences were observed between groups (mean ± SEM). **(D)** Behavioral hyperexcitability test. Notice a higher touch-response score in stimulated rats (SE, *n* = 15) at week 12 compared with week 6. *p < 0.05. The data are expressed as median and range (min, max).

All of the animal procedures were approved by the Ethical Committee on Animal Research of the Nencki Institute of Experimental Biology (permit no. 483/2013 of the Warsaw Local Ethics Committee for Animal Experimentation) and conducted in accordance with guidelines that were established by European Council Directive 2010/63/EU and accordance with the ARRIVE guidelines^8^.Twenty-six adult male Sprague–Dawley rats (270–300 g) from the Mossakowski Medical Research Centre, Polish Academy of Sciences (Warsaw, Poland), were used in this study. The experiments included two animal groups. The first group consisted of *n* = 8 control and *n* = 8 stimulated rats. The second group consisted of *n* = 4 control and *n* = 7 stimulated rats. The rats were housed under controlled conditions (24 °C, 50–60% humidity, 12 h/12 h light/dark cycle) with food and water available ad libitum. The animals were housed in pairs. Environmental enrichment was employed by using various toys (e.g., wooden and plastic balls) and snacks that were changed weekly. Starting 4 weeks before electrical stimulation, the rats were regularly handled every other day for 10 min. The timeline of the experiments is presented in Fig. 1A.

The amygdala stimulation model of temporal lobe epilepsy was used in the present study^9^. Status epilepticus was triggered by electrical stimulation of the amygdala as previously described^10^ with some modifications. Briefly, surgery was performed under isoflurane anesthesia (2–2.5% in 100% O_2_) that was preceded by an injection of butorphanol (Butomidor, Richter Pharma AG, Wells, Austria; 0.5 mg/kg, i.p.) for analgesia. Stimulating and recording bipolar electrodes (Plastic One, Roanoke, VA, USA; catalog no. E363-3-2WT-SPC) were implanted in the left lateral nucleus of the amygdala 3.6 mm posterior and 5.0 mm lateral to bregma, 6.5 mm ventral to the surface of the brain. A stainless-steel screw electrode (Plastics One, Roanoke, VA, USA; catalog no. E363/20) was implanted contralaterally in the skull over the right frontal cortex (3.0 mm anterior and 2.0 mm lateral to bregma) as a surface EEG recording electrode. Two stainless-steel screw electrodes were placed bilaterally over the cerebellum (10.0 mm posterior and 2.0 mm lateral to bregma) as ground and reference electrodes. The socket contacts of all of the electrodes were placed in a multi-channel electrode pedestal (Plastics One, Roanoke, VA, USA; catalog no. MS363) that was attached to the skull with dental acrylate (Duracryl Plus). After 2 weeks of recovery, the animals were electrically stimulated via the intra-amygdala electrode to evoke status epilepticus. Stimulation consisted of a 100-ms train of 1-ms biphasic square-wave pulses (400 μA peak to peak) that were delivered at 60 Hz every 0.5 s for 30 min. If the animal did not exhibit status epilepticus, then the stimulation was continued for an additional 10 min. Status epilepticus was stopped 1 h after the cessation of stimulation via an intraperitoneal injection of diazepam (20 mg/kg).If the first dose of diazepam did not suppress status epilepticus, then the animal received subsequent doses of 5 mg/kg diazepam. Time-matched control animals had electrodes implanted but did not receive electrical stimulation.

The rats were continuously monitored by video EEG (vEEG; Comet EEG, Grass Technologies, West Warwick, RI, USA) using a Panasonic WV-CP480 camera from the moment of stimulation until week 5 of the study (first vEEG recording), from week 11 to week 15 (second vEEG recording), and starting at week 27 until the end of the experiment (third vEEG recording; Fig. 1A). Spontaneous seizures were identified from the EEG recordings by browsing the EEG manually on the computer screen. An electrographic seizure was defined as a high-frequency (> 8 Hz) and high-amplitude (> 2 × baseline) discharge that lasted at least 5 s. The latency to the first spontaneous seizure, number and frequency of seizures, and number of epileptic animals in each group were evaluated. The analysis of video EEG showed that the stimulated animals developed partial and generalized seizures. Among the 15 stimulated animals, nine had at least one generalized seizure. However, the frequency of partial seizures was higher than generalized seizures. The number of generalized seizures was low: 83 of 3654 seizures (2.3%).

### Body weight monitoring

Body weight was monitored 2 weeks before and 0, 1, 2, 5, 9, 12, 24, and 31 weeks after stimulation (Fig. 1C). This parameter was used to assess the general health status of the rats after electrode implantation and at later time points.

### Behavioral tests

All of the animals in the study underwent a battery of behavioral tests, in the following order: open field test, novel object recognition test, elevated plus maze test (weeks 8 and 26 after the induction of status epilepticus) and Morris water maze test (weeks 9 and 27 after the induction of status epilepticus). All of the tests were recorded using WinTV software (Hauppauge, NY, USA). Video files were analyzed using EthoVision 8.5 software (Noldus, Leesburg, VA, USA), and the data were exported to Microsoft Excel. Tests were performed in an order from the least to the most stressful for the animals. By using different behavioral tests to assess similar parameters, such as anxiety or activity, we were able to validate the observed effect on these differenct parameters. Moreover, we could observe that a specific parameter was specifically sensitive in a particular test. To avoid negative effects of repeated testing which can influence the animal's activity all of the animals were familiarized with the testing areas prior to testing and we performed the behavioral tests beginning with the least invasive to the most invasive(and hence the least to the most stressful). The order of animals within trials was randomized.

The behavioral hyperexcitability test was performed at weeks 6 and 12 after stimulation as previously described^11^. Animal behavior was assessed in four categories: approach response, touch response, loud noise, and pick-up. In the approach-response test, a pen that was held vertically was moved slowly toward the face of the animal. Responses were scored as the following: 1 (no reaction), 2 (sniffing the pen), 3 (moving away from the pen), 4 (freezing), 5 (jumping away from the pen), and 6 (attacking the pen). In the touch-response test, the animal was gently prodded in the rump with the blunt end of the pen. Responses were scored as the following: 1 (no reaction), 2 (turning toward the touched area), 3 (moving forward, away from the touch), 4 (freezing), 5 (jerking around toward the touch), 6 (turning away from the touch), and 7 (jumping with or without vocalization). In the loud noise test, a clicking noise was generated by a timer, several centimeters above the head of the animal. Responses were scored as the following: 1 (no reaction), 2 (jumping slightly, flinching, or flicking the ears), and 3 (jumping abruptly). In the pick-up test, the animal was picked up by grasping it around the body. Responses were scored as the following: 1 (very easy), 2 (easy with vocalization), 3 (some difficulty, with the rat rearing and facing the experimenter’s hand), 4 (freezing with or without vocalization), 5 (difficult, with the rat avoiding the hand by moving away), and 6 (very difficult, with the rat behaving defensively, with or without attacking the experimenter’s hand). The behavioral hyperexcitability test was repeated four times during 1 day at 1 h intervals. Median scores were used for the subsequent data analysis.

The open field test and novel object exploration test were performed at weeks 8 and 26 using a square, dark gray box (1 m × 1 m) as previously described^12,13^. In the open field test, the animal was placed in the center of the arena. The rat’s movements were monitored for 30 min. The latency to enter the inner area of the arena, latency to enter the central area, and speed were monitored during the test. After 30 min of video monitoring animal was then removed from the arena and placed in a cage for 10 min while the arena was cleaned with 0.1% acetic acid and an object (black bottle, 2.5 cm diameter, 7 cm height) was placed in the center for the subsequent novel object exploration test. Novel object exploration test starts after 10 min break. The rat’s movements were monitored for 15 min. In novel object exploration test, the latency to enter the inner area of the arena, the latency to approach the novel object, and mobility were monitored.

The elevated plus maze test was performed at weeks 8 and 26 after stimulation. A crossed maze was elevated 50 cm above the floor, with two open arms and two closed arms. Each arm was 30 cm long and 5 cm wide, as previously described^14–16^. Briefly, the rat was placed in the central area of the maze and monitored for 5 min. The number of entries into the closed and open arms and speed were monitored during the test.

The Morris water maze test was performed at weeks 9 and 27 after stimulation using a swimming pool (2 m diameter, 1.5 m height) as previously described^9^. A platform (6 cm diameter, 50 cm height) was placed in one of four quadrants of the pool. The pool was filled with water (28 °C) to a depth of 2 cm above the platform. Visual cues (i.e., black circle and colorful poster) were placed on two opposite walls of the room. The rat’s behavior was recorded using an Ikegami ICD-505P camera that was placed above the pool, and EthoVision 3.0 software was used for recording and analysis. The data were exported to Microsoft Excel. The order of the animals was randomized within trials. The test consisted of 3 days of training (two trials, 60 s each). Memory was tested 48 h after the last training session. For the test, the platform was removed, and the animals were allowed to swim in the pool for 1 min. The time to locate the platform was measured during all training days. The time spent over the platform region, speed, the time spent in the target quadrant, and the time spent in the other quadrants were monitored during the test. Rodent Data Analysis (RODA) software was used to evaluate swimming strategies^17,18^.

### Morris water maze analysis using RODA software

EthoVision files from the Morris water maze test were analyzed using RODA 4.0.2 software^18^, which implements an updated classification procedure based on a previous study by Gehring et al.^17^. The same procedure was also applied by Huzard et al.^19^. For a detailed protocol, see: https://github.com/RodentDataAnalytics/mwm-ml-gen/wiki (accessed December 4, 2020). This analysis relies on categorizing the animals’ trajectories into classes of behaviors that are known as strategies that are implemented to solve the maze (Fig. 1B;^17,20^).

Briefly, the animal's trajectories inside the maze were segmented into overlapping segments 12% of which were manually labeled. RODA software was used to classify the remainder of the segments and map them back to the original trajectories. We used default tuning as described by Vouros et al. (2018) and the classification results were manually assessed^18^. In our analysis we used the following strategies: thigmotaxis (the animal moves along the periphery of the arena, close to the walls), incursion (the animal starts to move along the inward locations of the arena), scanning (the animal randomly searches the arena, preferably the central region, and turns away from the walls if it touches them), focused search (the animal actively searches a particular small region of the arena that is not where the platform is located), chaining response (the animal memorizes the distance of the platform from the arena wall and swims in a circular pattern to find it), self-orienting (the animal swims in a loop and orients itself inside the arena), scanning surroundings (the animal crosses the platform or a region very close to the platform), and scanning target (the animal actively searches near the platform location; Fig. 1B)^17,18^. The remaining segments are classified automatically. Raw data were exported to Microsoft Excel.

In a more detailed analysis behavioral strategies were grouped into low-level strategies and high-level strategies^18,19,21^. For example thigmotaxis and incursion were referenced as low-level strategies in Huzard et al. (2020) similar to the chaining response. However, such strategy groups are usually implemented based on specific experimental work. For example, in Huzard et al. (2020), the chaining response was listed as a low-level strategy in the sense that it is “sub-optimal” (i.e., the animal does not use spatial cues to learn the task, and its learning is adapted to a weak strategy). In the present study, thigmotaxis and incursion were considered low-level strategies because the animals mostly stayed in areas that were close to the walls of the arena. Scanning, focused search, chaining response, and self-orienting were classified as medium-level strategies because the animals explored inner parts of the arena. Scanning surroundings and scanning target were classified as high-level strategies because the animals passed or were focused on areas of the arena that contained the platform. We generally expected that impaired animals were likely to underuse medium- and high-level strategies or overuse low-level strategies. It would be informative to examine whether such behavioral differences are observed in epileptic animals.

### Perfusion

The rats were deeply anesthetized with isoflurane and perfused for histological analysis at week 32 after stimulation. Animals were perfused as previously described^9^ with some modifications. Sodium chloride (0.9%, kept on ice) was infused during first 20 min of perfusion followed by cold 4% paraformaldehyde (30 ml/min), pH 7.4 for the next 10–15 min. The brains were removed from the skull and incubated with 4% paraformaldehyde overnight. Next, the brains were cryoprotected in 30% saccharose in 0.02 M potassium phosphate buffered saline (KPBS, pH 7.4) for the next 48 h. The brains were then frozen in dry ice and stored at  − 80 °C. Brains were cryosectioned in the coronal plane and Nissl staining was performed to confirm electrode placements.

### Validation cohort

The animal procedure was approved by the Ethical Committee on Animal Research of the Nencki Institute (Permit No. 483/2013) at the Warsaw Local Ethics Committee for Animal Experimentation and conducted in accordance with the guidelines established by European Council Directive 2010/63/EU.

Thirteen adult male Sprague–Dawley rats (270–300 g) from Mossakowski Medical Research Centre Polish Academy of Sciences in Warsaw (Poland) were used as a validation cohort in this study. The validation cohort consisted of six sham animals and seven stimulated animals in which epilepsy was evoked using electrical stimulation of the amygdala as described above. Animals in the validation cohort were housed under environmentally enriched conditions with more toys and more frequent handling than the exploration group. All of the animals in the experiment underwent to the battery of behavioral tests including open field test, novel object exploration test, elevated plus maze test and Morris water maze test during weeks 8–9 after status epilepticus induction.

### Statistical analysis

The statistical analyses were performed using Prism 5 software (GraphPad, San Diego, CA, USA). Student’s *t*-test and repeated measures analysis of variance (ANOVA) followed by the Bonferroni post hoc test were used to analyze the Morris water maze and EEG data. One-way analysis of variance (ANOVA) followed by the Bonferroni post hoc test was used to analyze the other data. Values of *p* < 0.05 and *p* < 0.01 were considered statistically significant.

## Results

### Animals wellbeing

To investigate the animals’ general wellbeing, body weight was monitored during the study. The data are expressed as a percentage of body weight on the day of electrode implantation (i.e., 2 weeks before stimulation). No differences in percent body weight were found between the sham and stimulated animals at weeks 0–2 (Fig. 1C). No changes in body weight were observed between sham and stimulated rats at later time points after stimulation at weeks 5–31 (Fig. 1C).

### Influence of amygdala stimulation-induced status epilepticus on behavior

We did not observe any differences between sham and stimulated rats in the behavioral hyperexcitability test including approach response, touch response, loud noise, and pick-up (Fig. 1Dd1–d4). We observed a higher touch-response score in stimulated rats at week 12 compared with week 6 (median = 2, minimun = 1, maximum = 5 at week 6 vs. median = 3, mininimum = 1, maximum = 5 at week 12; *p* < 0.05; Fig. 1Dd2).

We did not detect differences in the open field test between sham and stimulated rats at weeks 8 and 26 including the latency to enter inner area of the arena, the latency to enter the central area, and speed (Supplementary Table S1A).

No changes were observed in the novel object exploration test between sham and stimulated rats at weeks 8 and 26 including the latency to enter the inner area of the arena, the latency to approach the novel object and mobility (Supplementary Table S1B).

We did not observe differences in the elevated plus maze test between sham and stimulated rats at weeks 8 and 26 including the number of entries into the closed arms, the number of entries into the open arms and speed (Supplementary Table S1C).

**Figure 2:**
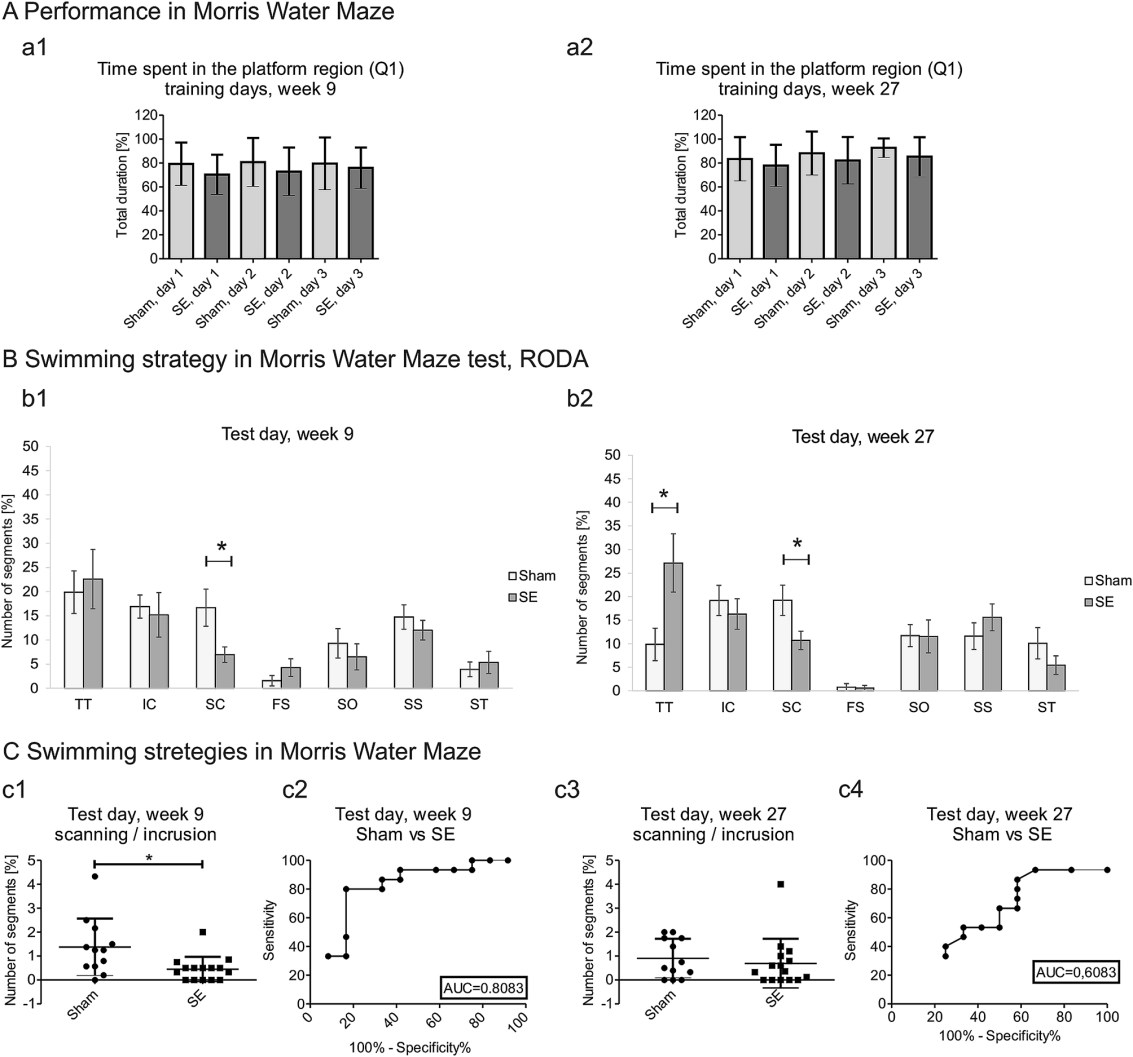
Performance in the Morris water maze test between sham and stimulated animals. **(A)** Time to find the platform (in seconds) in the Morris water maze during training days. No differences were observed at week 9 (**a**1) and week 27 (**a**2) (mean ± SD). (**B**) Analysis of swimming strategy in the Morris water maze on the test day using RODA software. The stimulated group (SE, *n* = 15) exhibited a decrease in the number of sections where they exhibited incursion (IC) and scanning (SC) strategies at weeks 9 and 27 of the experiment compared with sham animals (*n* = 12) (**b**1, **b**2). *p < 0.05. The focused search (FS) strategy increased at week 9, and thigmotaxis (TT) increased at week 27 in stimulated animals (SE, *n* = 15) compared with sham animals (*n* = 12). *p < 0.05 (mean ± SEM). (**C**) Comparison of scanning and incursion strategies in the Morris water maze. A lower proportion of scanning (SC) to incursion (IC) was observed in stimulated rats (SE, *n* = 15) compared with the sham group (*n* = 12) on the test day at week 9. *p < 0.05 (**c**1, **c**2). No differences were observed at week 27 (c3-c4) (mean ± SD).

No changes were observed in the Morris water maze between sham and stimulated rats at weeks 9 and 27 including the percent of time spent in the platform region (Q1) during 3 days of training (Fig. 2Aa1, Aa2), swimming time over the platform, speed, time spent in the target quadrant, time spent in quadrant 2, time spent in quadrant 3, and time spent in quadrant 4 (Supplementary Table S1D).

The principal component analysis of swimming strategies in the Morris water maze test did not separate sham and stimulated rats, indicating that performance in this test was not markedly disturbed by status epilepticus (Supplementary Fig. S2A). More detailed analyses of swimming strategies detected some specific impairments.

No significant differences in thigmotaxis, incursion, scanning, focused search, self-orienting, scanning surroundings or scanning target were observed during 3 days of training at week 9 or 27 following status epilepticus induction (Supplementary Table S3). The analysis of swimming strategies in the Morris water maze on the test day between sham and stimulated rats at weeks 9 and 27 showed no differences in thigmotaxis, incursion, self-orienting, scanning surroundings, or scanning target. Interestingly, we observed decreases in scanning at week 9 and week 27 (week 9: 16.6% ± 3.8% vs. 6.9% ± 1.6%, *p* < 0.05; week 27: 19.24% ± 3.2% vs. 10.7% ± 1.9%, *p* < 0.05) in the stimulation group compared with the sham group at both time points. We also observed increases in thigmotaxis at week 27 (9.8% ± 3.4% vs. 27.1% ± 6.1%, *p* < 0.05) in the stimulation group compared with the sham group (Fig. 2Bb1, Bb2). Moreover, a higher proportion of scanning to incursion was observed in the sham group compared with the stimulation group on the test day at week 9 (1.3% ± 1.2% vs. 0.4% ± 0.5%, *p* < 0.05; Fig. 2Cc1, Cc2) but not at week 27 (Fig. 2Cc3, Cc4).

More detailed analysis showed the transition probabilities among all of the strategies. The probabilities of strategy transitions were checked in the test trials (weeks 9 and 27) in the sham and stimulated groups (Supplementary Fig. S4). At week 9, the sham group transitioned more often from medium- to low-level strategies (57.4%) and from high- to medium-level strategies (63.2%) compared with the stimulated group (66.7% and 58.9%, respectively). The stimulated group also exhibited a slight preference for transitioning from low- to high-level strategies (59.0%) compared with the sham group (48.1%). At week 27, the same observations hold true for the sham group (transitions from high to medium increased to 81.5% and from medium to low to 62.2%) and the stimulated group (transitions from high to medium increased to 60.8% and from medium to low to 66.7%). The sham group also exhibited a higher probability of transitions from low- to medium-level strategies (71.1%) compared with week 9 (51.9%) and week 27 (45.5%) in the stimulated group. Overall, at week 27, the sham group exhibited a tendency toward transitioning to a medium strategy (81.5% from high to medium and 71.1% from low to medium), and the stimulated group exhibited a tendency toward transitioning to lower strategies (60.8% from high to medium and 66.7% from medium to low; Supplementary Fig. S4).

### Influence of speed of epilepsy development on behavior

To measure the speed of epilepsy development, we recorded the latency to the first spontaneous seizure. We divided the group of stimulated animals according to a median split of latency. This division resulted in groups of animals with a short latency (< 20 days, *n* = 7) and long latency (> 20 days, *n* = 8) to the first spontaneous seizure. We did not observe differences between the sham, short-latency, and long-latency groups in the behavioral hyperexcitability test including approach response, touch response, loud noise, and pick-up (Supplementary Table S5A).

No differences were observed in the open field test between the sham, short-latency, and long-latency groups at weeks 8 and 26 including the latency to enter the inner area of the arena, the latency to enter the central area, and speed (Supplementary Table S5B).

No differences were observed in the novel object exploration test between the sham, short-latency and long-latency groups at weeks 8 and 26 including the latency to enter the inner area of the arena, the latency to approach the novel object, and mobility (Supplementary Table S5C).

We did not observe changes in the elevated plus maze test between the sham, short-latency, and long-latency groups at weeks 8 and 26 including the number of entries into the closed arms, the number of entries into the open arms, and speed (Supplementary Table S5D).

**Figure 3:**
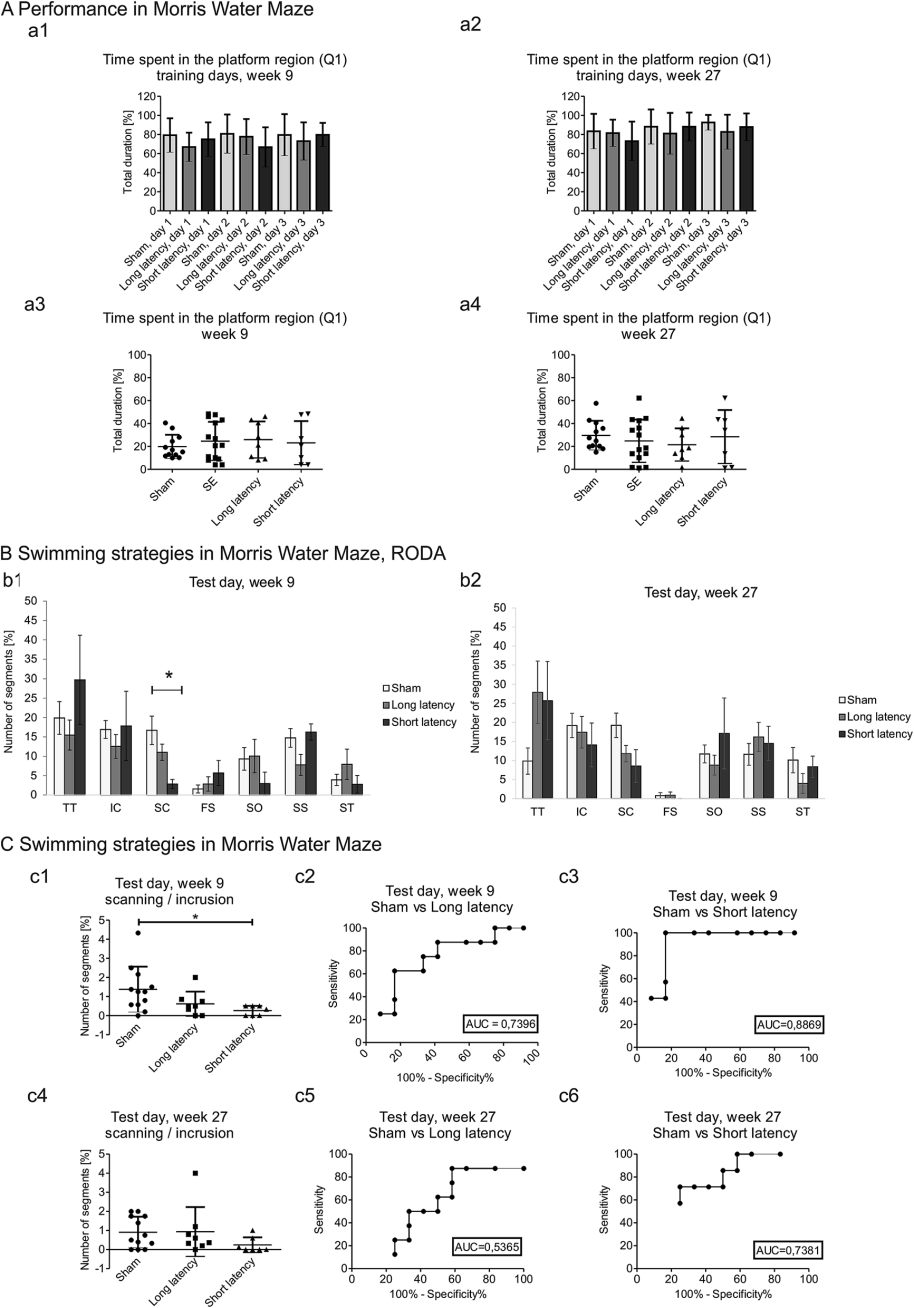
Performance and swimming strategies in the Morris water maze between sham, long-latency, and short-latency animals. (**A**) Time to find the platform and time spent in the target region. No differences were observed in the time to find the platform (in seconds) during Morris water maze training days at week 9 (**a**1) and week 27 (**a**3). No differences were observed in the time spent in the target region (Q1) at 9 week (**a**2) and week 27 (**a**4) between sham animals (*n* = 12), stimulated animals (including long- and short-latency animals together; SE, *n* = 15), the long-latency group (*n* = 8), and the short-latency group (*n* = 7) (mean ± SD). (**B**) Analysis of swimming strategy in the Morris water maze on the test day using RODA software. The short-latency group (*n* = 7) exhibited a decrease in the number of sections where they presented a scanning (SC) strategy at week 9 of the experiment compared with sham animals (*n* = 12) (b1). *p < 0.05. No differences were observed between the sham, long-latency, and short-latency groups at week 27 (**c**2) (mean ± SEM). (**C**) Comparison of scanning and incursion strategies in the Morris water maze. A higher proportion of scanning (SC) to incursion (IC) was observed in the sham group (*n* = 12) compared with the short-latency group (*n* = 7) on the test day of the Morris water maze test at week 9 (*p < 0.05) (**c**1, **c**3) and week 27 (*p < 0.05) (**c**4, **c**6). No differences were observed between the sham (*n* = 12) and long-latency (*n* = 8) groups at week 9 (**c**1, **c**2) and week 27 (**c**4, **c**5) (mean ± SD).

No differences were observed in the Morris water maze between the sham, short-latency, and long-latency groups at weeks 9 and 27 including the percent of time spent in the target region (Q1) during 3 days of training (Fig. 3Aa1, Aa2), swimming time over the platform, speed, time spent in the target quadrant (Fig. 3Aa3, Aa4), time spent in quadrant 2, time spent in quadrant 3, and time spent in quadrant 4 (Supplementary Table S5E).

The principal component analysis of swimming strategies in the Morris water maze test did not separate the sham, short-latency and long-latency groups, indicating that performance in this test was not markedly disturbed by status epilepticus (Supplementary Fig. S2B). More detailed analysis detected some specific impairments.

No significant differences in thigmotaxis, incursion, scanning, focused search, self-orienting, scanning surroundings, or scanning target were observed during 3 days of training at week 9 or 27 following the induction of status epilepticus (Supplementary Table S6).

Interestingly, the analysis of swimming strategies in the Morris water maze test detected a decrease in scanning strategy in the short-latency and long-latency groups compared with the sham group at week 9 (16.6% ± 3.8% vs. 2.8% ± 1.0% vs. 11.0% ± 4.1%, respectively; *p* < 0.05;Fig. 3Bb1). No differences in the following strategies were observed between groups at week 27 during the test day including thigmotaxis, incursion, scanning, focused search, self-orienting, scanning surroundings, and scanning target (Fig. 3Bb1, Bb2). Moreover, a lower proportion of scanning to incursion was observed in the short-latency group compared with the sham group during the test day at week 9 (1.3% ± 1.1% vs. 0.2% ± 0.2% vs. 0.7% ± 0.6%, respectively; *p* < 0.05; Fig. 3Cc1–3).No differences in proportion of scanning to incrusion between sham, short latency and long latency groups were observed 27 week (Fig. 3Cc4–6). We observed a correlation between latency to the first spontaneous seizure versus scanning strategy at week 9 after stimulation (R =  − 0.7088; *p* < 0.01).

More detailed analyses were conducted to determine the transition probabilities among all strategies. The probabilities of strategy transitions were checked in test trials (weeks 9 and 27) in the sham, short-latency, and long-latency groups (Supplementary Fig. S7). At week 9, the probabilities of transitions in the sham group were 51.9% from low- to medium-level, 57.4% from medium- to low-level and 63.2% from high- to medium-level. In the long-latency group, the probabilities were 58.3% from low- to high-level strategies, 66.7% from medium- to low-level strategies, and 78.6% from high- to medium-level strategies. In the short-latency group, the probabilities were 59.5% from low- to high-level strategies, 66.7% from medium- to low-level strategies, and 60.7% from high- to low-level strategies. At week 27, the probabilities in the sham group were 81.5% from high- to medium-level strategies, 62.2% from medium- to low-level strategies, and 71.1% from low- to medium-level strategies. In the long-latency group, the probabilities were 57.1% from low- to medium-level strategies, 74.1% from medium- to low-level strategies, and 69.3% from high- to medium-level strategies. In the short-latency group, the probabilities were 75.0% from low- to high-level strategies, 55.6% from medium- to low-level strategies, and 54.2% from high- to low-level strategies.

### Influence of epilepsy on behavior

To examine whether epilepsy influences behavior, we divided the cohort of stimulated animals into two groups: stimulated rats that developed spontaneous seizures (*n* = 7) and stimulated rats that did not develop spontaneous seizures (*n* = 8) before the first behavioral tests. We did not observe differences between the sham, non-epileptic, and epileptic groups in the behavioral hyperexcitability test including approach, touch response, loud noise, and pick-up (Supplementary Table S8A).

We did not detect changes in the open field test between the sham, non-epileptic, and epileptic groups at week 8 including the latency to enter the inner area of the arena, the latency to enter the central area, and speed (Supplementary Table S8B).

No differences were observed in the novel object exploration test between the sham, non-epileptic, and epileptic groups at week 8 including the latency to enter the inner area of the arena, the latency to approach the novel object, and mobility (Supplementary Table S8C).

We did not observe differences in the elevated plus maze test between the sham, non-epileptic, and epileptic groups at week 8 including the number of entries into the closed arms, the number of entries into the open arms, and speed (Supplementary Table S8D).

**Figure 4:**
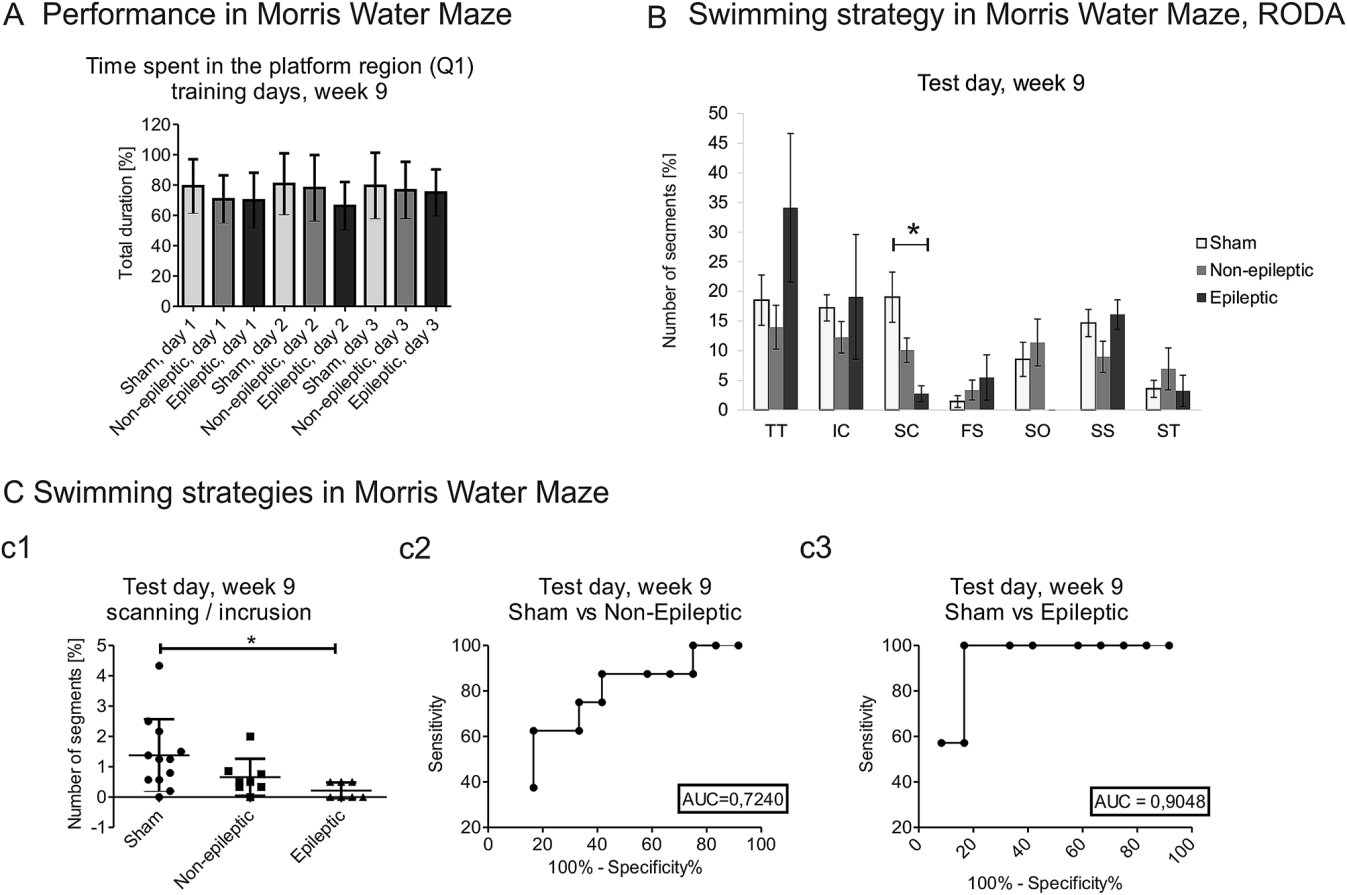
Performance and swimming strategies in the Morris water maze test in sham, non-epileptic, and epileptic animals at week 9. (**A**) Performance in the Morris water maze test. No differences were observed in the time to find the platform (in seconds) during Morris water maze training days at week 9 of the experiment between sham (*n* = 12), non-epileptic (*n* = 8), and epileptic (*n* = 7) animals (**a**1) (mean ± SD). (**B**) Analysis of swimming strategy on the Morris water maze test day using RODA software. The epileptic group (*n* = 7) exhibited a decrease in the number of sections where they presented a scanning (SC) strategy at week 9 compared with the sham group (*n* = 12). *p < 0.05 (mean ± SEM). (**C**) Comparison of scanning and incursion strategies in the Morris water maze. A higher proportion of scanning (SC) to incursion (IC) was observed in the sham group (*n* = 12) compared with the epileptic group (*n* = 7) at week 9. *p < 0.05 (**c**1, **c**3). No differences were observed between sham and non-epileptic animals (*n* = 8) (**c**1, **c**2) (mean ± SD).

No changes in the percent time spent in the target region (Q1)were observed in the Morris water maze test between the sham, non-epileptic, and epileptic groups at week 9 during 3 days of training (Fig. 4A).

We did not detect differences in Morris water maze performance between the sham, non-epileptic, and epileptic groups at week 9 including swimming time over the platform, speed, time spent in the target quadrant, time spent in quadrant 2, time spent in quadrant 3, and time spent in quadrant 4 (Supplementary Table S8E).

We found significant differences in swimming strategies in the Morris water maze. The epileptic group exhibited an increase in thigmotaxis compared with the non-epileptic group at week 9 in the second trial during the second day of training (day 2, trial 2: 10.7% ± 7.8% vs. 3.7% ± 8.3%, *p* < 0.05; Supplementary Table S9). No differences in thigmotaxis were found between the sham, non-epileptic, and epileptic groups during the first day, first trial on the second day, and third day of the Morris water maze training days. No significant differences in incursion, scanning, focused search, self-orienting, scanning surroundings, or scanning target were observed between the sham, non-epileptic, and epileptic groups during 3 days of training at week 9 following status epilepticus induction (Supplementary Table S9). The analysis of swimming strategies in the Morris water maze on the test day detected a decrease in scanning in the epileptic group compared with the sham group at week 9 (16.6% ± 3.8% vs. 2.7% ± 1.3%, *p* < 0.05). No differences were observed in thigmotaxis, incursion, focused search, self-orienting, scanning surroundings, and scanning target (Fig. 4B). A higher proportion of scanning to incursion was observed in the epileptic group compared with the sham group during the test day at week 9 (1.3% ± 1.2% vs. 0.6% ± 0.6% vs. 0.2% ± 0.2%, respectively; *p* < 0.05; Fig. 4Cc1–c3).

More detailed analyses showed the probabilities of transitions among strategies in test trials at week 9 after stimulation in the sham, non-epileptic, and epileptic groups (Supplementary Fig. S10). In the sham group, the probabilities were 51.9% from low- to medium-level strategies, 57.4% from medium- to low-level strategies, and 63.2% from high to medium**-**level strategies. In the non-epileptic group, the probabilities were 50% to both the probabilities from low- to medium and to high-level strategies, 66.7% from medium- to low-level strategies, and 75.0% from high- to medium**-**level strategies. In the epileptic group, the probabilities were 69.4% from low- to high-level strategies, 66.7% from medium- to low-level strategies, and 62.5% from high- to low-level strategies.

### Influence of epilepsy intensity on behavior

As a measure of the intensity of epilepsy development, we recorded the total number of spontaneous seizures. We divided the group of stimulated animals according to a median split of the total number of spontaneous seizures, resulting in a group with a low number of seizures (62 ± 64.5, *n* = 8) and a group with a high number of seizures (456 ± 185.0, *n* = 7).

We did not observe any differences between the sham, low-seizure-number, and high-seizure-number groups in the behavioral hyperexcitability test including approach response, touch response, loud noise, and pick-up (Supplementary Table S11A).

No differences were detected in the open field test between the sham, low-seizure-number, and high-seizure-number groups at weeks 8 and 26 including the latency to enter the inner area of the arena, the latency to enter the central area, and speed (Supplementary Table S11B).

No differences were observed in the novel object exploration test between the sham, low-seizure-number, and high-seizure-number groups at weeks 8 and 26 including the latency to enter the inner area of the arena, the latency to approach the novel object, and mobility (Supplementary Table S11C).

**Figure 5:**
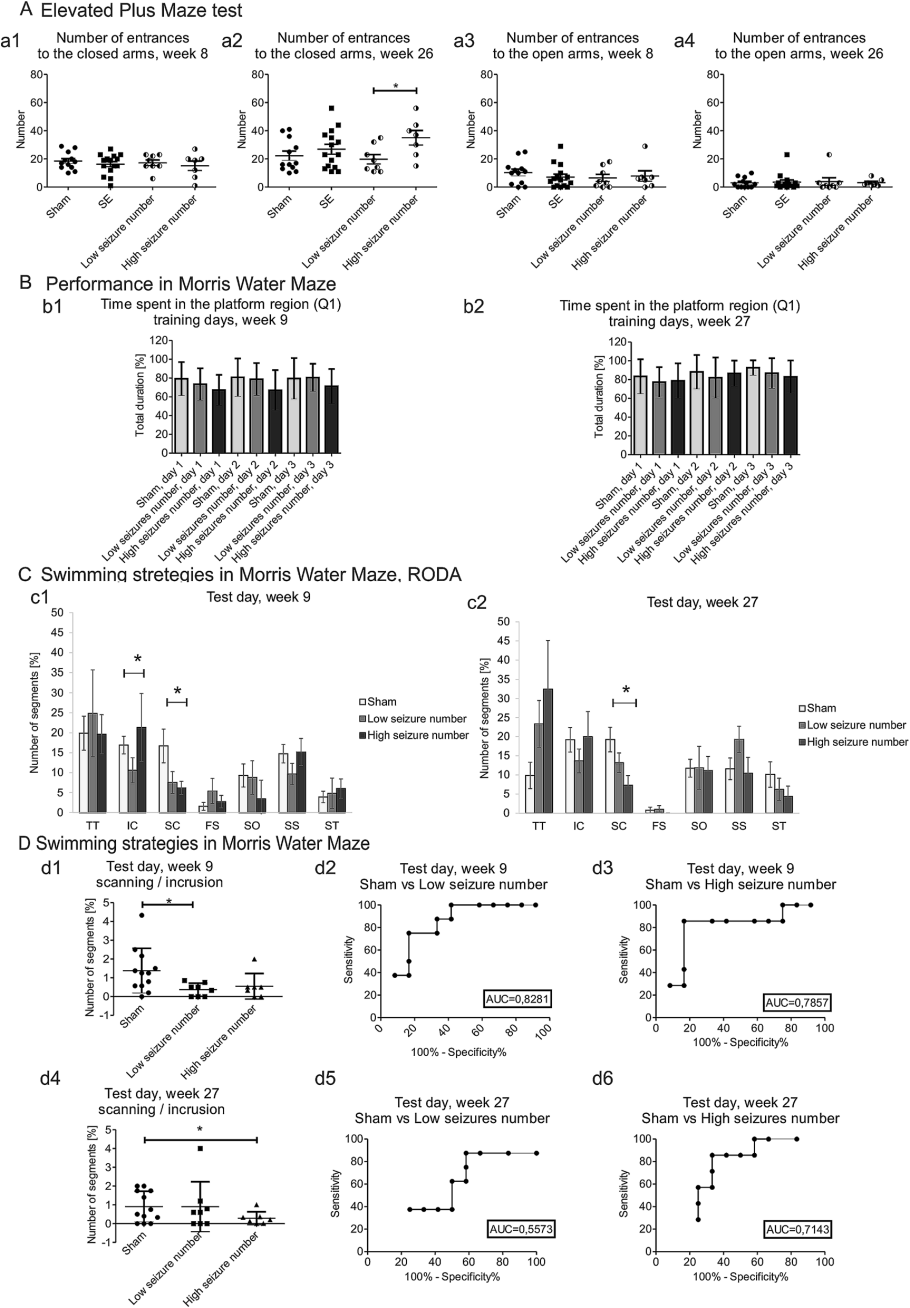
Performance between sham, low-seizure-number, and high-seizure-number groups in the elevated plus maze test and Morris water maze test (with swimming strategy analysis) 9 and 27 weeks after stimulation. (**A**) Performance in the elevated plus maze test. The high-seizure-number group (*n* = 8) exhibited an increase in the number of entries into the closed arms in the elevated plus maze test at week 26 compared with the low-seizure-number group (**a**2). *p < 0.05. No differences were observed between groups in the number of entries into the closed arms at week 8 (**a**1) or the number of entries into the open arms at weeks 8 and 26 of the experiment (**a**3, **a**4) (mean ± SEM). (**B**) Performance in the Morris water maze. No differences were observed in the time to find the platform (in seconds) during Morris water maze training days at week 9 (**b**1) and week 27 (**b**2) of the experiment between the sham (*n* = 12), low-seizure-number (*n* = 7), and high-seizure-number (*n* = 8) groups (mean ± SEM). (**C**) Analysis of swimming strategy on the Morris water maze test day using RODA software. The high-seizure-number group (*n* = 8) exhibited an increase in the number of sections where they presented an incursion (IC) strategy compared with sham animals (**c**1). *p < 0.05. The high-seizure-number group (*n* = 8) exhibited a decrease in the scanning (SC) strategy at week 9 and week 27 of the experiment compared with the sham group (*n* = 12) (**c**1, **c**2). *p < 0.05 (mean ± SEM). (**D**) Comparison of scanning and incursion strategies in the Morris water maze. A higher proportion of scanning (SC) to incursion (IC) was observed in the sham group (*n* = 12) compared with the low-seizure-number group (*n* = 7) on the Morris water maze test day at week 9 (**d**1, **d**2). *p < 0.05. The high-seizure-number group exhibited a lower proportion of scanning (SC) to incursion (IC) compared with the sham group (*n* = 12) on the Morris water maze test day at week 27 (d4, d6). *p < 0.05. No differences were observed between the sham (*n* = 12) and high-seizure-number (*n* = 8) groups at week 9 (**d**1, **d**3) or between the sham (*n* = 12) and low-seizure-number (*n* = 7) groups at week 27 of the experiment (d4-d5) (mean ± SD).

No differences were observed in the elevated plus maze test between the sham, epileptic, low-seizure-number, and high-seizure-number groups at week 8 including the number of entries into the closed arms (Fig. 5Aa1). Interestingly, we detected an increase in the number of entries into the closed arms in the high-seizure-number group compared with the low-seizure-number group at week 26 (19.7 ± 3.3 vs. 35.0 ± 5.1, respectively; *p* < 0.05; Fig. 5Aa2). No differences were found between the sham, epileptic, low-seizure-number, and high-seizure-number groups at weeks 8 and 26 including the number of entries into the open arms (Fig. 5Aa3, Aa4) and speed (Supplementary Table S11D).

No differences were observed in the Morris water maze between the sham, low-seizure-number, and high-seizure-number groups at weeks 9 and 27including the percent time spent in the target region (Q1) during 3 days of training (Fig. 5Bb1, Bb2).

No differences were observed in the Morris water maze test between the sham, low-seizure-number, and high-seizure-number groups at weeks 9 and 27 including swimming time over the platform, speed, time spent in the target quadrant, time spent in quadrant 2 (, time spent in quadrant 3, and time spent in quadrant 4 (Supplementary Table S11E).

The principal component analysis of swimming strategies in the Morris water maze did not separate the sham, low-seizure-number and high-seizure-number groups, indicating that performance in this test was not markedly disturbed by status epilepticus (Supplementary Fig. S2C). A more detailed analysis of swimming strategies detected some specific impairments.

No significant differences in thigmotaxis, incursion, scanning, focused search, self-orienting, scanning surroundings, or scanning target were found between the sham, low-seizure-number, and high-seizure-number groups during 3 days of training at week 9 or 27 following status epilepticus induction (Supplementary Table S12).

Interestingly, the analysis of swimming strategies in the Morris water maze test detected an increase in incursion in the high-seizure-number group compared with the sham group at week 9 (16.9% ± 2.4% vs. 21.3% ± 8.5%, *p* < 0.05; Fig. 5Cc1). A decrease in scanning was observed in the high-seizure-number group compared with the sham group at both time points (week 9: 16.6% ± 3.8% vs. 6.1% ± 1.6%, *p* < 0.05; week 27: 19.2% ± 3.2% vs. 11.9% ± 2.6%, *p* < 0.05; Fig. 5Cc1, Cc2). No differences were observed in thigmotaxis, incursion, focused search, self-orienting, scanning surroundings or scanning target (Fig. 5Cc1, Cc2). Moreover, a lower proportion of scanning to incursion was observed in the low-seizure-number group compared with the sham group during the test day at week 9 (1.3% ± 1.1% vs. 0.3% ± 0.3%, *p* < 0.05; Fig. 5Dd1–3). Interestingly, a lower proportion of scanning to incursion in the Morris water maze test day was observed in the high-seizure-number group compared with the sham group at week 27 (0.9% ± 0.8% vs. 0.3% ± 0.3%, *p* < 0.05; Fig. 5Dd4–6). We observed a correlation between the number of seizures and scanning strategy at week 9 after stimulation (R =  − 0.5322; *p* < 0.05).

More detailed analyses were conducted to determine transition probabilities among all of the strategies. Transition probabilities were checked in the test trials (weeks 9 and 27) in the sham, low-seizure-number, and high-seizure-number groups (Supplementary Fig. S13). At week 9, the probabilities in the sham group were 51.9% from low- to medium-level strategies, 57.4% from medium- to low-level strategies, and 63.2% from high- to medium-level strategies. In the low-seizure-number group, the probabilities were to both the probabilities from low- to medium- and low- to high-level strategies, 66.7% from medium to low-level strategies, and 75.0% from high- to medium**-**level strategies. In the high-seizure-number group, the probabilities were 69.4% from low- to high-level strategies, 66.7% from medium- to low-level strategies, and 62.5% from high- to low**-**level strategies. At week 27, the probabilities in the sham group were 81.5% from high-to medium-level strategies, 62.2% from medium- to low-level strategies, and 71.1% from low- to medium-level strategies. In the low-seizure-number group, the probabilities were 57.1% from low- to medium-level strategies, 58.3% from medium to low-level strategies, and 55.0% from high- to medium-level strategies. In the low-seizure-number group, the probabilities were 75.0% from low- to high-level strategies, 79.2% from medium- to low-level strategies, and 70.8% from high- to medium-level strategies.

### Validation cohort

To examine predictive validity of the behavioral measures we performed a battery of behavioral tests using a validation cohort that consisted of six sham and seven stimulated animals that were housed under environmentally enriched conditions.

We did not detect changes in the open field test between the sham and stimulated groups at week 8 including the latency to enter the inner area of the arena, the latency to enter the central area, and speed (Supplementary Table S14A).

No differences were observed in the novel object exploration test between the sham and stimulated groups at week 8 including the latency to enter the inner area of the arena, the latency to approach the novel object, and mobility (Supplementary Table S14B).

We did not observe differences in the elevated plus maze test between the sham and stimulated groups at week 8 including the number of entries into the closed arms, the number of entries into the open arms, and speed (Supplementary Table S14C).

No differences were observed in Morris water maze performance between sham and stimulated groups at week 9 including swimming time over the platform, speed, time spent in the target quadrant (4), time spent in quadrant 1, time spent in quadrant 2, and time spent in quadrant 3 (Supplementary Table S14D).

We did not observe differences in thigmotaxis, incursion, scanning, focused search, self-orienting or scanning surroundings in Morris water maze test between the sham and stimulated groups. However, in the validation cohort we observed a trend toward an increased thigmotaxis in the stimulated group compared with the sham group. Stimulated animals also exhibited a trend toward an decreased in scanning strategy. Interestingly, we observed a significant decrease in the stimulated group compared with the sham animals (17.2 ± 12.0 vs. 4.7 ± 6.0; *p* < 0.05; Supplementary Figure S15).

## Discussion

The present study investigated the possibility of using common behavioral tests as a source of biomarkers for use in preclinical studies in the epilepsy field. We were interested in identifying behavioral indices that can differentiate epileptic and non-epileptic animals and disease phenotypes. We identified only a few, limited specific behavioral disturbances that could distinguish these groups. We observed differences between sham and epileptic animals (but not between stimulated epileptic and stimulated non-epileptic animals) in swimming strategies during the test session in the Morris water maze and behavioral hyperexcitability test. We also found that epileptic animals that had a high number of seizures differed from epileptic animals that had a low number of seizures in the number of entries into the closed arms of the elevated plus maze.

In the present study we performed a battery of behavioral tests, in the following order: behavioral hyperexcitability test (weeks 6 and 12 after status epilepticus induction), open field test, novel object recognition test, elevated plus maze test (weeks 8 and 26 after status epilepticus induction) and Morris water maze test (weeks 9 and 27 after status epilepticus induction). Tests were performed in an order from the least to the most stressful for the animals. Repeated testing can have different impacts on performance in behavioral tests depending on the treatment group which is unavoidable. Our testing order began from less invasive (and the least stressful) to the most invasive (and thus most stressful). Animal's performance was not severely influenced by repeated testing effect because we saw few differences between groups.

### Comparison between sham and stimulated animals

We used a well-characterized model of temporal lobe epilepsy in rats in which epilepsy is evoked by amygdala stimulation^9,10,22–24^. Behavioral deficits in the Morris water maze have been reported in stimulated animals in this model^9^, but the present study was the first to conduct a comprehensive panel of behavioral tests to search for noninvasive behavioral biomarkers.

Behavioral and cognitive impairments that result from status epilepticus have been observed in many rat models of epilepsy. Rats that were subjected to pilocarpine-induced epilepsy exhibited an increase in depressive-like behavior in a post-seizure behavioral test battery (also called the behavioral hyperexcitability test) 3–4 weeks after status epilepticus induction^11^. The strongest reaction was observed in the touch-response and pick-up tests. Rattka et al. (2013) reported that kainate-treated rats had significantly higher scores in the touch-response and finger-snap tests than sham-treated controls^25^. Higher scores in the touch-response test were also observed between epileptic and sham rats 11–12 weeks after status epilepticus induction^26^. Similarly, our data showed higher scores in the touch-response test in stimulated animals 12 weeks after epilepsy induction, suggesting that rats with seizures had a higher level of anxiety-like behavior than the sham group.

Nissinen et al. (2000) observed spatial learning impairments in epileptic animals in the Morris water maze. Epileptic animals exhibited impairments in the ability to find the platform compared with control animals during all 7 days of training^9^. Similarly, Rattka et al. reported that kainate-treated rats had a significantly longer escape latency during days 3 and 4 of training compared with sham-treated controls 30 weeks after status epilepticus induction^25^. Inostroza et al. (2011) also reported a longer escape latency in kainate-treated rats at 3 days of the experiment, whereas lithium + pilocarpine-treated rats had a longer escape latency at 2 and 3 days of training, 8 weeks after epilepsy induction^27^. In the present study, no changes in escape latency were observed during any of the training days at weeks 9 and 27 of the experiment, which suggests that the stimulated animals were not impaired or had significantly less impairment in spatial learning. This may be explained by the fact that behavioral tests are sensitive to specific experimental and environmental conditions, including housing conditions^28–33^. Notably, our animals were housed under environmental enrichment conditions, in contrast to the previous studies mentioned above.

To analyze Morris water maze performance more closely, we used RODA software to quantitatively analyze the rats’ learning strategies^17^. We observed different swimming strategies in epileptic animals, and their swimming strategies differed according to the intensity of epilepsy and latency to the first spontaneous seizure. Previous studies of epilepsy reported behavioral impairments that were caused by epilepsy and epileptogenesis, mainly reflected by measures of thigmotaxis in the Morris Water Maze test. In the present study, we performed a more detailed analysis of swimming strategies and found that thigmotaxis in the Morris water maze was indicative of behavioral and learning impairments. Using the RODA software, we analyzed different swimming strategies and evaluated differences between different types of strategies. The analysis of different swimming patterns showed specific significant changes in swimming strategies. The sham group spent more time in the central area of the pool, exhibiting incursion and scanning strategies. Both of these swimming patterns represent a place strategy and maze exploration, meaning that the rats actively searched inward areas of the arena because the platform was located there and moved away from the wall. Stimulated animals spent more time near the wall at week 27 of the experiment and exhibited a thigmotaxis strategy, which was previously reported in epileptic animals^34^. The behavior of these rats may suggest place navigation disability or anxiety-like behavior. The receiver operating characteristic analysis of the ratio between scanning and incursion strategies showed that the stimulated group spent more time in the outer area of the arena, whereas the sham group spent more time in the inner area, looking for the platform. As per our expectations, stimulated animals underused medium-level strategies and overused low-level strategies (see Fig. 2, week 27). Sham animals at both weeks 9 and 27 transitioned slightly more often from a medium-level strategy to a high-level strategy and less often from a high-level strategy to a low-level strategy compared with the stimulated group. Based on strategy transitions from low to medium levels and from low to high levels, little difference was observed in stimulated animals between weeks 9 and 27, whereas sham animals had a higher probability of ending at a medium-level strategy at week 27. These results showed that sham animals had a higher probability of making better decisions to find the platform than stimulated animals.

### Comparison between sham, long latency and short latency groups

Nissinen et al. (2000) used the same animal model of temporal lobe epilepsy and found that stimulated animals had a variable latency to the first spontaneous seizure, which oscillated around week 1 and weeks 2–3 after stimulation^9^. Little is known about behavioral differences between rats that have short versus long latencies to develop spontaneous seizures.

We found that alterations of swimming strategies in the Morris water maze depended on the latency to the first spontaneous seizure. In the standard analysis of the Morris water maze data, we did not observe any significant differences between sham animals, stimulated animals with a long latency to spontaneous seizures, and stimulated animals with a short latency to spontaneous seizure during all 3 days of training. No differences were found between groups in the percent time spent in the platform region during the test day. The analysis of swimming strategies showed that animals with a short latency to the first spontaneous seizure exhibited a decrease in the scanning strategy compared with sham animals at week 9 of the experiment. Sham-operated animals spent more time exploring the maze and searching for the platform region than animals with a short latency.

The ratio between scanning and incursion strategies showed that animals with a short latency to the first spontaneous seizure spent more time in the outer area of the arena at week 9. Overall and similarly to our previous observation, stimulated animals underuse medium level strategies and overuse low level strategies (see Fig. 2 week 27). These results indicate that the rats’ behavioral strategies in the Morris water maze were related to the latency to the first spontaneous seizure.

### Comparison between sham, non-epileptic and epileptic groups

We observed behavioral alterations in the Morris water maze in stimulated epileptic animals compared with sham and stimulated non-epileptic animals at week 9 after stimulation. The scanning strategy in epileptic animals significantly decreased compared with sham animals. Moreover, the ratio between the scanning and incursion strategies indicates that epileptic animals spent more time in the outer area of the arena, whereas sham animals spent more time searching for the platform. These results suggest that spontaneous seizures affected the rats’ place navigation strategy.

### Comparison between sham, low seizures number and high seizures number groups

Nissinen et al. (2000) used the same animal model of temporal lobe epilepsy and found that epileptic animals could be divided into subgroups of animals with low and high seizure frequencies. They found that epileptic rats’ swimming navigation depended on seizure frequency. Their data showed that animals with fewer seizures found the platform faster than animals with more seizures^9^. In the present study, the low-seizure-number and high-seizure-number groups did not differ from the sham group during all training days of the Morris water maze test at weeks 9 and 27 after stimulation. The analysis of swimming strategy showed that animals with a high number of seizures exhibited an increase in incursion and a decrease in scanning compared with sham animals. These results show that the rats’ place navigation strategy depended on seizure frequency. The receiver operating characteristic analysis of the ratio between scanning and incursion indicated differences between the sham and high-seizure-number groups, indicating that sham rats spent more time engaged in a scanning strategy at week 27 of the experiment compared with the high-seizure-number group. Interestingly, the ratio between scanning and incursion at week 9 of the experiment significantly decreased in the low-seizure-number group compared with the sham group. These results indicate that even a low number of spontaneous seizures affected the place navigation strategy. Strategy transitions (refer to Supplementary Fig. S13) also revealed that behavioural transitions probabilities of the low seizure number group on week 27 are more similar to the behavioural transitions probabilities of the sham group on week 9 which are more equally distributed over all the strategies indicating that seizures frequency can reduce the animal behaviors back to their original state. We detected impairments in motor activity in the elevated plus maze. Previous findings in pilocarpine-treated mice did not detect behavioral impairments in the elevated plus maze, and motor activity was not different between the pilocarpine-treated group and controls^35^. However, Langer et al. (2011) showed that stimulated rats exhibited a significant increase in the total distance travelled and spent less time on the open arms of the elevated plus maze than controls^36^. Our data showed that stimulated animals did not differ from the sham group in the elevated plus maze test. We did not observe differences between animals with a short latency and animals with a long latency to the first spontaneous seizure at weeks 8 and 26 after stimulation. The elevated plus maze test showed no differences between the sham, low-seizure number and high-seizure number groups at week 8 after stimulation. However, the high-seizure number group exhibited an increase in the number of entries into the closed arms compared with the low-seizure number group at week 26. These results suggest that seizure frequency can influence hyperactivity in the elevated plus maze test.

### Comparison between exploration cohort and validation cohort

Similar results were observed in the exploration cohort and validation cohort. No differences were observed between sham and stimulated animals from the exploration and validation cohorts in the open field test, novel object exploration test, and elevated plus maze test.

The standard analysis of the Morris Water Maze data showed no differences between sham and stimulated animals in the exploration cohort and validation cohort during the test at week 9. We also performed an analysis of swimming strategy using RODA software. Interestingly, in the validation cohort sham animals exhibited a significant increase in scanning target compared with stimulated animals. These results showed that sham animals learned the localization of the platform. In the validation cohort, we also observed a trend toward an increase in thigmotaxis in the stimulated group compared with sham animals. Moreover, stimulated animals exhibited a trend toward a decrease in scanning strategy but an increase in scanning target strategy, meaning that they spent more time looking for the platform. These findings are similar to the data that were obtained from the exploration cohort.

## Conclusions

In conclusion, higher motor activity in the elevated plus maze may be a potential noninvasive biomarker of the epileptic phenotype. Our study showed that the intensity of epilepsy and duration of epileptogenesis influenced swimming strategies in the Morris water maze test increasing low-level strategies where the animal navigates close to the walls of the arena. The analysis of different swimming strategies might help identify epileptic animals and may also be used as a noninvasive biomarker in preclinical studies.

## Supplementary Information

Supplementary Information.

## References

[1] Duncan JS, Sander JW, Sisodiya SM, Walker MC (2006). Adult epilepsy. Lancet.

[2] Galanopoulou AS (2012). Identification of new epilepsy treatments: issues in preclinical methodology. Epilepsia.

[3] Pitkanen A, Lukasiuk K (2011). Mechanisms of epileptogenesis and potential treatment targets. Lancet Neurol..

[4] Klee R, Brandt C, Tollner K, Loscher W (2017). Various modifications of the intrahippocampal kainate model of mesial temporal lobe epilepsy in rats fail to resolve the marked rat-to-mouse differences in type and frequency of spontaneous seizures in this model. Epilepsy Behav..

[5] Pitkanen A, Sutula TP (2002). Is epilepsy a progressive disorder? Prospects for new therapeutic approaches in temporal-lobe epilepsy. Lancet Neurol..

[6] Pitkanen A, Lukasiuk K, Dudek FE, Staley KJ (2015). Epileptogenesis. Cold Spring Harbor Perspect. Med..

[7] Kandel ER, Shawartz JH, Jessell TM, Siegelbaum SA, Hudspeth AJ (2013). Principles of Neural Science.

[8] Kilkenny C, Browne WJ, Cuthill IC, Emerson M, Altman DG (2010). Improving bioscience research reporting: the ARRIVE guidelines for reporting animal research. Plos Biol..

[9] Nissinen J, Halonen T, Koivisto E, Pitkanen A (2000). A new model of chronic temporal lobe epilepsy induced by electrical stimulation of the amygdala in rat. Epilepsy Res..

[10] Guzik-Kornacka A, Sliwa A, Plucinska G, Lukasiuk K (2011). Status epilepticus evokes prolonged increase in the expression of CCL3 and CCL4 mRNA and protein in the rat brain. Acta Neurobiol. Exp..

[11] Rice AC, Floyd CL, Lyeth DG, Hamm RJ, DeLorenzo RJ (1998). Status epilepticus causes long-term NMDA receptor-dependent behavioral changes and cognitive deficits. Epilepsia.

[12] Gould TD, Dao DT, Kovacsics CE (2009). The open field test. Mood Anxiety Relat. Phenotypes Mice Charact. Using Behav. Tests.

[13] Antunes M, Biala G (2012). The novel object recognition memory: neurobiology, test procedure, and its modifications. Cogn. Process..

[14] Kiryk A (2011). Transient brain ischemia due to cardiac arrest causes irreversible long-lasting cognitive injury. Behav. Brain Res..

[15] Walf AA, Frye CA (2007). The use of the elevated plus maze as an assay of anxiety-related behavior in rodents. Nat. Protoc..

[16] Hogg S (1996). A review of the validity and variability of the elevated plus-maze as an animal model of anxiety. Pharmacol. Biochem. Behav..

[17] Gehring TV, Luksys G, Sandi C, Vasilaki E (2015). Detailed classification of swimming paths in the Morris water maze: multiple strategies within one trial. Sci. Rep..

[18] Vouros A (2018). A generalised framework for detailed classification of swimming paths inside the Morris water maze. Sci. Rep..

[19] Huzard D (2020). Constitutive differences in glucocorticoid responsiveness are related to divergent spatial information processing abilities. Stress.

[20] Dalm S, Grootendorst J, de Kloet ER, Oitzl MS (2000). Quantification of swim patterns in the Morris water maze. Behav. Res. Methods Instrum. Comput..

[21] Janus C (2004). Search strategies used by APP transgenic mice during navigation in the Morris water maze. Learn Mem..

[22] Bednarczyk J, Debski KJ, Bot AM, Lukasiuk K (2016). MBD3 expression and DNA binding patterns are altered in a rat model of temporal lobe epilepsy. Sci. Rep..

[23] Sliwa A, Plucinska G, Bednarczyk J, Lukasiuk K (2012). Post-treatment with rapamycin does not prevent epileptogenesis in the amygdala stimulation model of temporal lobe epilepsy. Neurosci. Lett..

[24] Pitkanen A (2002). Progression of neuronal damage after status epilepticus and during spontaneous seizures in a rat model of temporal lobe epilepsy. Do Seizures Damage Brain.

[25] Rattka M, Brandt C, Loscher W (2013). The intrahippocampal kainate model of temporal lobe epilepsy revisited: epileptogenesis, behavioral and cognitive alterations, pharmacological response, and hippoccampal damage in epileptic rats. Epilepsy Res..

[26] Brandt C, Gastens AM, Sun MZ, Hausknecht M, Loscher W (2006). Treatment with valproate after status epilepticus: effect on neuronal damage, epileptogenesis, and behavioral alterations in rats. Neuropharmacology.

[27] Inostroza M (2011). Hippocampal-dependent spatial memory in the water maze is preserved in an experimental model of temporal lobe epilepsy in rats. PLoS ONE.

[28] Dersi G (2016). Environmental enrichment imparts disease-modifying and transgenerational effects on genetically-determined epilepsy and anxiety. Neurobiol. Dis..

[29] Faverjon S (2002). Beneficial effects of enriched environment following status epilepticus in immature rats. Neurology.

[30] Koh S, Chung H, Xia HJ, Mahadevia A, Song YJ (2005). Environmental enrichment reverses the impaired exploratory behavior and altered gene expression induced by early-life seizures. J. Child Neurol..

[31] Vrinda M (2017). Enriched environment attenuates behavioral seizuresand depression in chronictemporal lobe epilepsy. Epilepsia.

[32] Wang CA (2007). An enriched environment improves cognitive performance after early-life status epilepticus accompanied by an increase in phosphorylation of extracellular signal-regulated kinase 2. Epilepsy Behav..

[33] Yang M (2016). Environmental enrichment delays limbic epileptogenesis and restricts pathologic synaptic plasticity. Epilepsia.

[34] Lee CL (2001). Spatial learning deficits without hippocampal neuronal loss in a model of early-onset epilepsy. Neuroscience.

[35] Groticke I, Hoffmann K, Loscher W (2007). Behavioral alterations in the pilocarpine model of temporal lobe epilepsy in mice. Exp. Neurol..

[36] Langer M, Brandt C, Loscher W (2011). Marked strain and substrain differences in induction of status epilepticus and subsequent development of neurodegeneration, epilepsy, and behavioral alterations in rats Strain and substrain differences in an epilepsy model in rats. Epilepsy Res..

