# Supplementary material for: Behavioral characteristics as potential biomarkers of the development and phenotype of epilepsy in a rat model of temporal lobe epilepsy
Source: Sci Rep. 2021 Apr 21;11:8665. doi: 10.1038/s41598-021-88088-9 (PMC8060252; doi:10.1038/s41598-021-88088-9)

Behavioral characteristics as potential biomarkers of the development and phenotype of epilepsy in a rat model of temporal lobe epilepsy

Karolina Nizińska[1]*, Kinga Szydłowska[1], Avgoustinos Vouros[2], Anna Kiryk[1], Aleksandra Stępniak[1], Eleni Vasilaki[2], Katarzyna Łukasiuk[1]

[1] Laboratory of Epileptogenesis, Nencki Institute of Experimental Biology, Warsaw, Poland

[2] Department of Computer Science, Faculty of Engineering, University of Sheffield, Great Britain

* Correspondence author, e-mail: k.nizinska@nencki.edu.pl

Supplementary Information

Supplementary Table S1. Behavioral changes between the sham and stimulated animals (Mean±SD).

A

Open field test	Week 8			Week 26		
**Type:**	Sham	SE	p value	Sham	SE	p value
**latency to enter inner area of the arena [s]**	40.2 ± 24.7	24.3 ± 9.0	p > 0.05	199.4 ± 108.7	58.6 ± 25.2	p > 0.05
**latency to enter the central area [s]**	142.8 ± 36.6	138.6 ± 57.8	p > 0.05	394.7 ± 131.8	141.6 ± 38.15	p > 0.05
**speed [cm/s]**	4.6 ± 0.4	5.6 ± 0.4	p > 0.05	4.4 ± 0.4	4.4 ± 0.4	p > 0.05

B

Novel object exploration test	Week 8			Week 26		
**Type:**	Sham	SE	p value	Sham	SE	p value
**latency to enter the inner area of the arena [s]**	59.0 ± 48.3	24.8 ± 11.0	p > 0.05	11.2 ± 1.7	12.0 ± 3.4	p > 0.05
**latency to approach the novel object [s]**	63.6 ± 48.3	30.9 ± 10.7	p > 0.05	12.6 ± 1.8	16.9 ± 3.5	p > 0.05
**mobility [s]**	54.4 ± 18.4	86.7 ± 36.7	p > 0.05	133.9 ± 23.8	197.2 ± 23.8	p > 0.05

C

Elevated plus maze test	Week 8			Week 26		
**Type:**	Sham	SE	p value	Sham	SE	p value
**number of entries into the closed arms**	17.7 ± 1.7	16.2 ± 1.8	p > 0.05	22.2 ± 3.0	26.8 ± 3.5	p > 0.05
**number of entries into the open arms**	10.6 ± 2.2	7.1 ± 2.1	p > 0.05	3.0 ± 0.9	3.5 ± 1.4	p > 0.05
**speed [cm/s]**	6.1 ± 0.3	6.0 ± 0.5	p > 0.05	4.1 ± 0.2	4.3 ± 0.2	p > 0.05

D

Morris water maze test	Week 9			Week 27		
**Type:**	Sham	SE	p value	Sham	SE	p value
**swimming time over the platform [s]**	1.9 ± 0.2	1.7 ± 0.3	p > 0.05	2.3 ± 0.2	1.86 ± 0.3	p > 0.05
**speed [cm/s]**	31.2 ± 1.1	30.8 ± 1.0	p > 0.05	31.9 ± 1.0	32.6 ± 1.3	p > 0.05
**time spent in the target quadrant [s]**	19.7 ± 3.01	26.0 ± 3.9	p > 0.05	26.6 ± 3.6	26.4 ± 4.8	p > 0.05
**time spent in quadrant 2 [s]**	19.7 ± 1.8	20.0 ± 2.2	p > 0.05	25.5 ± 2.6	19.7 ± 2.8	p > 0.05
**time spent in quadrant 3 [s]**	14.3 ± 1.1	11.2 ± 1.5	p > 0.05	13.8 ± 1.3	11.9 ± 1.4	p > 0.05
**time spent in quadrant 4 [s]**	10.3 ± 3.5	11.6 ± 2.3	p > 0.05	17.4 ± 1.8	17.8 ± 3.5	p > 0.05

Supplementary Figure S2. Swimming strategy in Morris water maze at weeks 9 and 27 after stimulation, PCA analysis

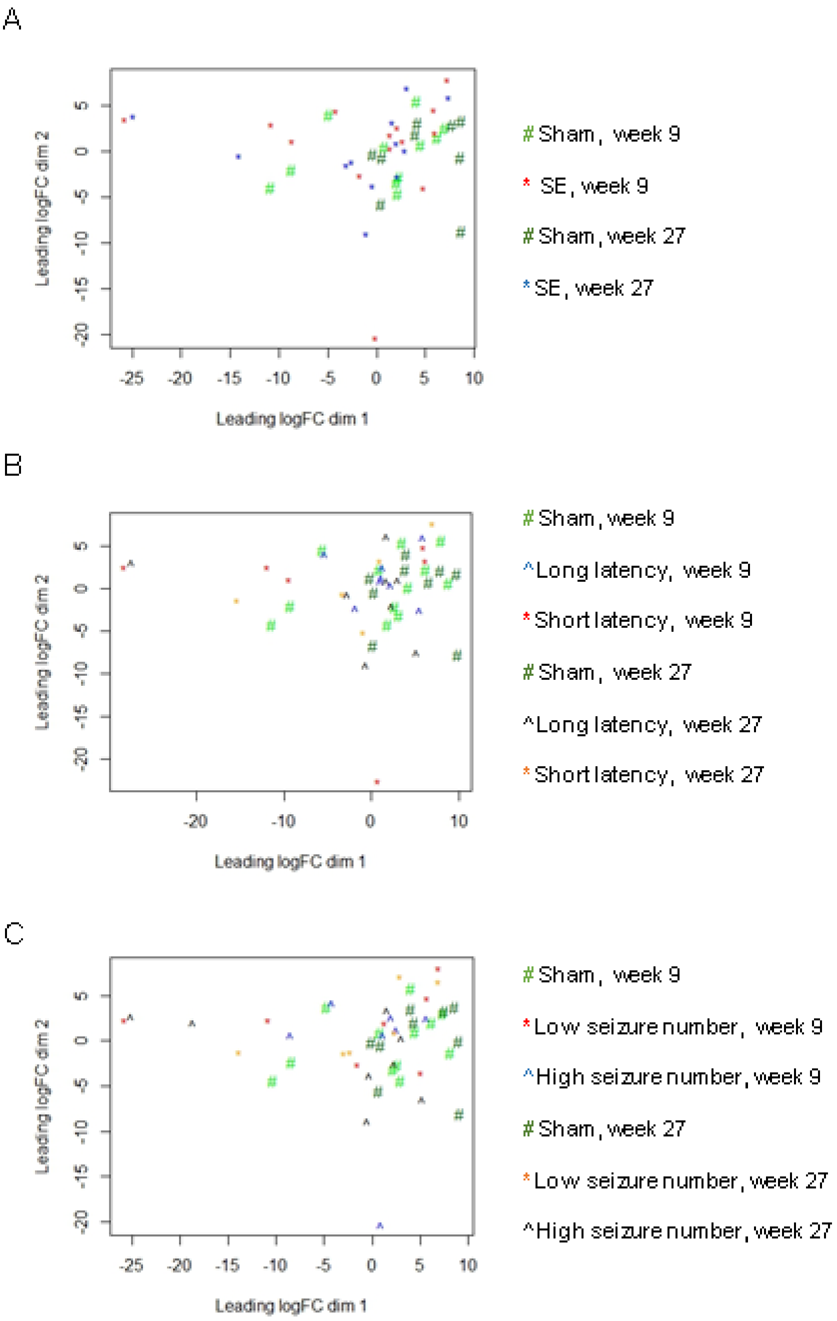

Swimming strategy (TT: thigmotaxis, IC: incursion, SC: scanning, FS: focused search, CR: chaining reaction, SO: self-orienting, SS: scanning surrounding, ST: scanning target) in Morris Water Maze at weeks 9 and 27 after stimulation between (A) sham vs stimulated (SE) animals, (B) sham vs long latency group vs short latency group , (C) sham vs low seizures number group vs high seizures number group.

Supplementary Table S3. Comparison at RODA analysis of Morris water maze training between the sham and stimulated animals at weeks 9 and 27 after stimulation (Mean±SD).

**RODA analysis of Morris Water Maze Training**			**Week 9**			**Week 27**		
**Type:**	Day:	Trial:	Sham [%]	SE [%]	p value	Sham [%]	SE [%]	p value
**Thigmotaxis**	day 1	1	12.0±16.4	57.0±21.5	p > 0.05	4.2±4.7	8.1±13.6	p > 0.05
		2	2.0±4.1	16.6±25.7	p > 0.05	0±0	3.2±8.6	p > 0.05
	day 2	1	25.0±15.2	25.0±28.7	p > 0.05	0.3±1.1	13.0±23.8	p > 0.05
		2	8.1±10.2	8.6±8.4	p > 0.05	0±0	2.3±7.1	p > 0.05
	day 3	1	13.8±16.9	17.1±28.6	p > 0.05	1.3±3.1	16.1±30.5	p > 0.05
		2	1.5±3.3	4.6±12.3	p > 0.05	3.8±5.3	7.4±18.9	p > 0.05
**Incrusion**	day 1	1	7.1±8.4	12.2±9.0	p > 0.05	5.2±6.0	7.2±9.8	p > 0.05
		2	6.9±6.2	8.0±4.8	p > 0.05	2.9±4.4	7.3±6.2	p > 0.05
	day 2	1	10.2±4.8	15.6±7.7	p > 0.05	3.3±4.6	8.4±7.7	p > 0.05
		2	3.7±5.5	6.2±10.2	p > 0.05	0±0	5.1±8.9	p > 0.05
	day 3	1	16.0±17.0	6.9±8.5	p > 0.05	4.2±7.3	5.9±5.2	p > 0.05
		2	4.2±6.8	3.7±5.7	p > 0.05	3.5±4.3	4.8±6.5	p > 0.05
**Scanning**	day 1	1	19.3±1.5	0.4±3.2	p > 0.05	6.9±6.3	7.3±5.0	p > 0.05
		2	6.2±5.3	4.6±9.0	p > 0.05	4.7±3.6	8.5±9.1	p > 0.05
	day 2	1	6.5±6.6	7.9±7.9	p > 0.05	9.5±8.6	7.2±7.9	p > 0.05
		2	3.1±4.4	4.5±3.9	p > 0.05	4.9±1.7	7.8±7.1	p > 0.05
	day 3	1	6.2±3.4	8.4±6.9	p > 0.05	4.9±5.3	8.1±7.0	p > 0.05
		2	10.1±8.6	8.7±8.7	p > 0.05	1.9±2.8	5.2±6.4	p > 0.05
**Focused search**	day 1	1	22.2±3.1	1.0±1.6	p > 0.05	2.3±4.3	6.4±9.0	p > 0.05
		2	0±0	0.5±1.2	p > 0.05	1.4±4.3	4.0±5.3	p > 0.05
	day 2	1	2.8±4.0	1.4±3.6	p > 0.05	3.9±5.2	1.3±4.6	p > 0.05
		2	1.8±4.1	1.0±6.3	p > 0.05	0.8±1.7	0.0±0	p > 0.05
	day 3	1	0±1.1	1.7±4.6	p > 0.05	1.0±2.3	1.6±3.5	p > 0.05
		2	2.3±6.6	2.1±6.2	p > 0.05	0±0	0±0	p > 0.05
**Self-orienting**	day 1	1	26.1±0	0.8±2.1	p > 0.05	7.6±8.6	7.9±9.5	p > 0.05
		2	2.7±4.2	3.1±3.9	p > 0.05	3.0±4.8	4.8±5.1	p > 0.05
	day 2	1	0.5±1.8	1.9±3.8	p > 0.05	7.1±6.5	6.8±11.1	p > 0.05
		2	3.7±8.3	5.5±6.2	p > 0.05	0.8±1.6	0.7±2.1	p > 0.05
	day 3	1	2.2±3.4	1.5±3.2	p > 0.05	1.6±2.3	0±0	p > 0.05
		2	5.0±6.6	3.4±6.5	p > 0.05	1.4±3.1	0±0	p > 0.05
**Scanning surroundings**	day 1	1	32.2±8.0	6.0±6.8	p > 0.05	7.0±8.7	3.1±4.8	p > 0.05
		2	3.8± 4.0	6.2±6.3	p > 0.05	1.4±2.3	5.1±6.4	p > 0.05
	day 2	1	4.2±4.2	4.8±4.4	p > 0.05	5.5±8.6	7.7±12.6	p > 0.05
		2	5±4.7	6.5±7.9	p > 0.05	5.9±4.4	2.2±4.5	p > 0.05
	day 3	1	4.4±5.9	2.9±6.1	p > 0.05	3.3±3.9	5.7±11.6	p > 0.05
		2	1.1±2.3	3.7±6.1	p > 0.05	3.4±4.9	3.9±5.2	p > 0.05
**Scanning target**	day 1	1	40.3±2.7	0.6±2.4	p > 0.05	4.1±7.6	2.2±5.9	p > 0.05
		2	5±9.0	5.6±7.8	p > 0.05	6.6±8.5	3.8±7.6	p > 0.05
	day 2	1	1.6±5.7	3.0±7.2	p > 0.05	8.3±9.3	2.5±3.8	p > 0.05
		2	14.2±8.7	9.6±9.5	p > 0.05	12.7±10.0	5.5±8.9	p > 0.05
	day 3	1	9.3±9.6	1.3±5.3	p > 0.05	1.8±6.0	5.2±7.6	p > 0.05
		2	6.6±9.8	7.0±9.7	p > 0.05	11.6±10.2	8.8±8.3	p > 0.05

Supplementary Figure S4 Comparison of RODA analysis of Morris water maze test day between the sham and stimulated animals at weeks 9 and 27 after stimulation.

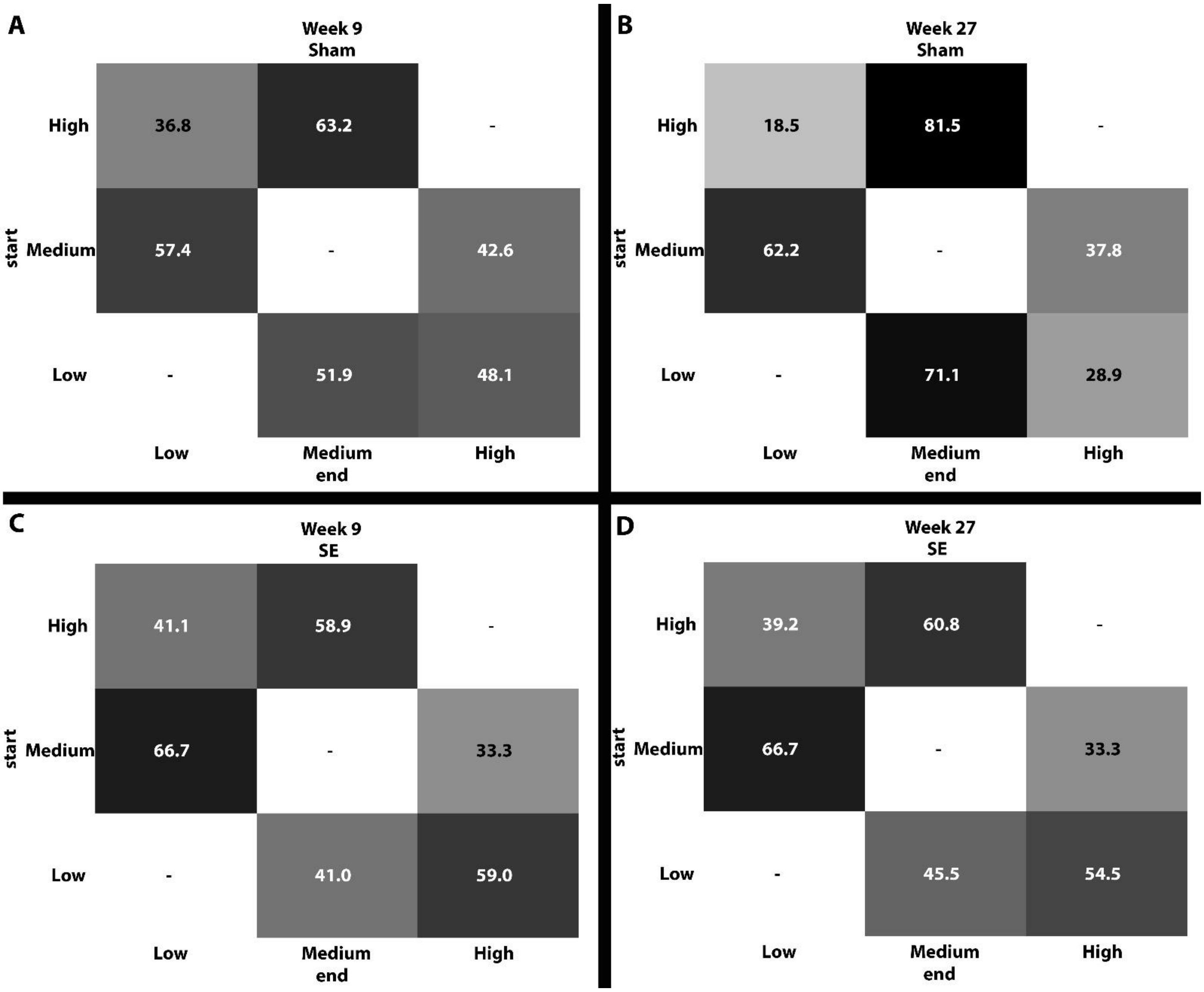

The probabilities (%) of transitioning between strategies were checked in the test trials (weeks 9 and 27) in the sham animals and stimulated (SE) groups. Rows and columns indicate the starting and ending strategies respectively. Row values (for the same starting strategy) were normalized (sum of each row equals 100%). Low strategies: thigmotaxis, incursion. Medium strategies: scanning, focused search, chaining reaction, self-orienting. High strategies: scanning surrounding, scanning target. Thigmotaxis and incursion were assigned to low-level strategies because the animals are staying mostly in the areas close to the walls of the arena. Scanning, focused search, chaining response and self-orienting were assigned to medium-level strategies because the animals explored inner parts of the arena. Scanning surroundings and scanning target were assigned to high-level strategies because the animals passed or focused on areas of the arena contained the platform. Sham animals at both weeks 9 and 27 transitioned more often from a medium-level to a high-level strategy and less often from a high-level to a low-level strategy than the SE group. Based on the strategy transitions from low to medium levels and low to high levels, little difference was seen in SE animals between weeks 9 and 27, whereas the sham animals were more likely to end with a medium-level strategy at week 27.

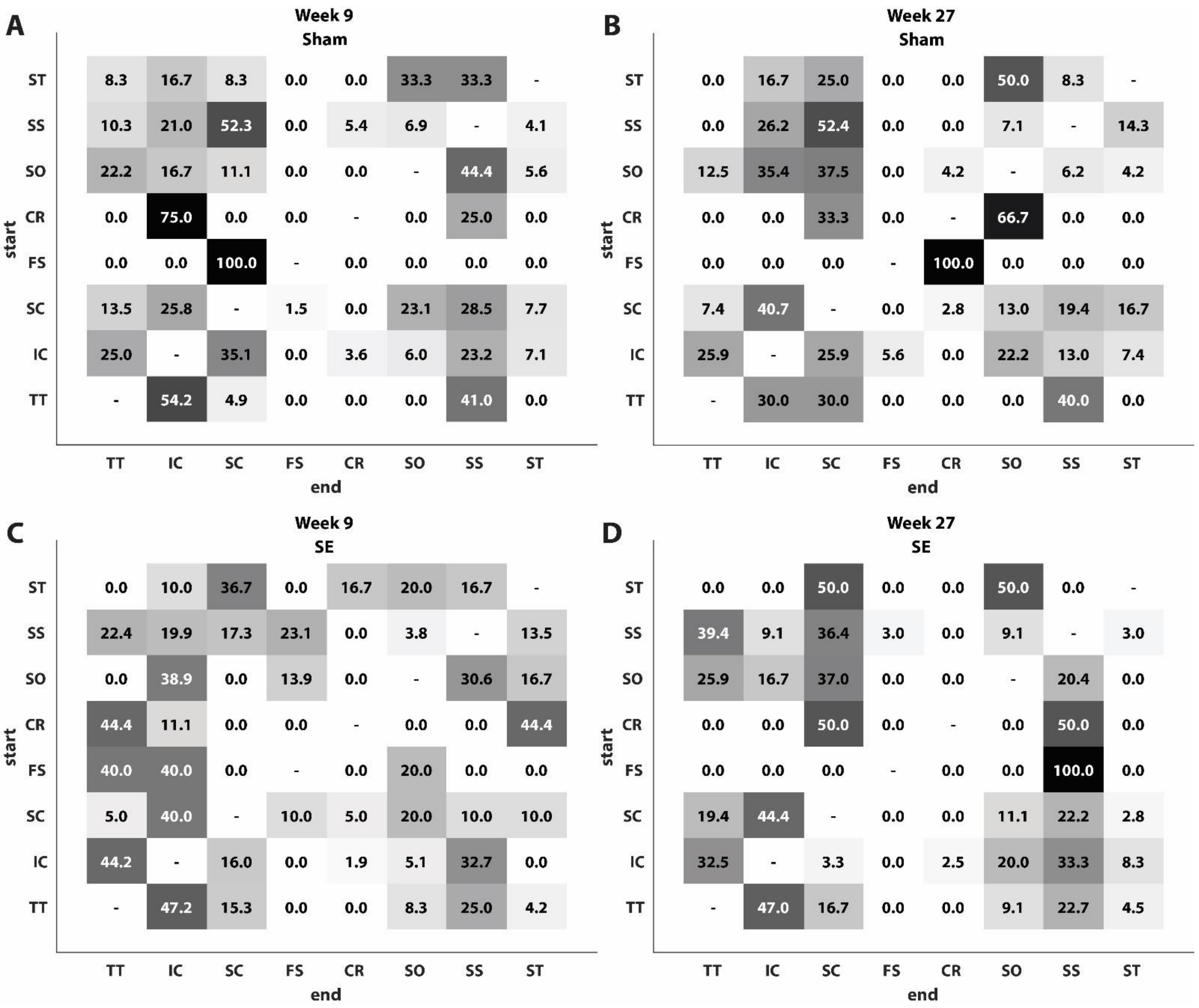
A
Week 9
Sham
ST 8.3 16.7 8.3 0.0 0.0 33.3 33.3 -
SS 10.3 21.0 52.3 0.0 5.4 6.9 - 4.1
SO 22.2 16.7 11.1 0.0 0.0 - 44.4 5.6
CR 0.0 75.0 0.0 0.0 - 0.0 25.0 0.0
FS 0.0 0.0 100.0 - 0.0 0.0 0.0 0.0
SC 13.5 25.8 - 1.5 0.0 23.1 28.5 7.7
IC 25.0 - 35.1 0.0 3.6 6.0 23.2 7.1
TT - 54.2 4.9 0.0 0.0 0.0 41.0 0.0
start
TT IC SC FS CR SO SS ST
end
B
Week 27
Sham
ST 0.0 16.7 25.0 0.0 0.0 50.0 8.3 -
SS 0.0 26.2 52.4 0.0 0.0 7.1 - 14.3
SO 12.5 35.4 37.5 0.0 4.2 - 6.2 4.2
CR 0.0 0.0 33.3 0.0 - 66.7 0.0 0.0
FS 0.0 0.0 0.0 - 100.0 0.0 0.0 0.0
SC 7.4 40.7 - 0.0 2.8 13.0 19.4 16.7
IC 25.9 - 25.9 5.6 0.0 22.2 13.0 7.4
TT - 30.0 30.0 0.0 0.0 0.0 40.0 0.0
start
TT IC SC FS CR SO SS ST
end
C
Week 9
SE
ST 0.0 10.0 36.7 0.0 16.7 20.0 16.7 -
SS 22.4 19.9 17.3 23.1 0.0 3.8 - 13.5
SO 0.0 38.9 0.0 13.9 0.0 - 30.6 16.7
CR 44.4 11.1 0.0 0.0 - 0.0 0.0 44.4
FS 40.0 40.0 0.0 - 0.0 20.0 0.0 0.0
SC 5.0 40.0 - 10.0 5.0 20.0 10.0 10.0
IC 44.2 - 16.0 0.0 1.9 5.1 32.7 0.0
TT - 47.2 15.3 0.0 0.0 8.3 25.0 4.2
start
TT IC SC FS CR SO SS ST
end
D
Week 27
SE
ST 0.0 0.0 50.0 0.0 0.0 50.0 0.0 -
SS 39.4 9.1 36.4 3.0 0.0 9.1 - 3.0
SO 25.9 16.7 37.0 0.0 0.0 - 20.4 0.0
CR 0.0 0.0 50.0 0.0 - 0.0 50.0 0.0
FS 0.0 0.0 0.0 - 0.0 0.0 100.0 0.0
SC 19.4 44.4 - 0.0 0.0 11.1 22.2 2.8
IC 32.5 - 3.3 0.0 2.5 20.0 33.3 8.3
TT - 47.0 16.7 0.0 0.0 9.1 22.7 4.5
start
TT IC SC FS CR SO SS ST
end

Supplementary Table S5. Behavioral changes between the sham, short latency and long latency groups (Mean±SD).

A

Behavioral hyperexcitability test	Week 6 [median (range: min; max)]				Week 12 [median (range: min; max)]			
**Type:**	Sham	Short latency	Long latency	p value	Sham	Short latency	Long latency	p value
**approach response**	2 (1;4)	2 (1;4)	2 (1;3)	p > 0.05	2 (1;4)	2 (1;4)	2 (1;4)	p > 0.05
**touch response**	2 (1;3)	2 (1;5)	2 (1;5)	p > 0.05	2 (1;4)	2 (1;5)	3 (1;5)	p > 0.05
**loud noise**	2 (1;3)	2 (1;3)	2 (1;2)	p > 0.05	2 (1;3)	2 (1;2)	2 (1;3)	p > 0.05
**pick-up**	1 (1;5)	2 (1;5)	1 (1;5)	p > 0.05	1 (1;5)	1 (1;5)	1 (1;5)	p > 0.05

B

Open field test	Week 8				Week 26			
**Type:**	Sham	Short latency	Long latency	p value	Sham	Short latency	Long latency	p value
**latency to enter inner area of the arena [s]**	40 ± 24.7	30.9 ± 16.1	19.4 ± 11.7	p > 0.05	119.4 ± 108.7	49.1 ± 43.9	68.1 ± 33.3	p > 0.05
**latency to enter the central area [s]**	142.8 ± 36.6	77.9 ± 77.9	184.2 ± 89.9	p > 0.05	394.7 ± 131.8	67.53 ± 55.7	197.17 ± 48.7	p > 0.05
**speed [cm/s]**	4.6 ± 0.4	5.9 ± 0.7	5.3 ± 0.5	p > 0.05	4.4 ± 0.4	4.7 ± 0.6	5.1 ± 0.5	p > 0.05

C

Novel object exploration test	Week 8				Week 26			
**Type:**	Sham	Short latency	Long latency	p value	Sham	Short latency	Long latency	p value
**latency to enter the inner area of the arena [s]**	59.0 ± 48.3	37.2 ± 18.7	7.6 ± 3.2	p > 0.05	11.2 ± 1.7	12.7 ± 4.9	10.2 ± 5.8	p > 0.05
**latency to approach the novel object [s]**	63.6 ± 48.3	43.9 ± 20.8	15.3 ± 6.5	p > 0.05	12.6 ± 1.8	18.7 ± 5.2	15.2 ± 6.4	p > 0.05
**mobility [s]**	54.4 ± 18.4	79.7 ± 54.2	93.0 ± 53.9	p > 0.05	133.9 ± 23.8	146.1 ± 26.2	248.3 ± 30.0	p > 0.05

D

Elevated plus maze test	Week 8				Week 26			
**Type:**	Sham	Short latency	Long latency	p value	Sham	Short latency	Long latency	p value
**number of entries into the closed arms**	17.7 ± 1.7	17.0 ± 2.2	15.6 ± 3.0	p > 0.05	22.2 ± 3.0	31.1 ± 5.6	23.1 ± 4.3	p > 0.05
**number of entries into the open arms**	10.6 ± 2.2	5.7 ± 2.0	8.3 ± 3.6	p > 0.05	3.0 ± 0.9	5.1 ± 3.0	2.2 ± 0.9	p > 0.05
**speed [cm/s]**	6.1 ± 0.3	5.7 ± 0.9	6.3 ± 0.8	p > 0.05	4.1 ± 0.2	4.3 ± 0.3	4.3 ± 0.3	p > 0.05

E

Morris water maze test	Week 9				Week 27			
**Type:**	Sham	Short latency	Long latency	p value	Sham	Short latency	Long latency	p value
**swimming time over the platform [s]**	1.9 ± 0.2	2.0 ± 0.7	1.4 ± 0.2	p > 0.05	2.3 ± 0.2	2.4 ± 0.5	1.2 ± 0.4	p > 0.05
**speed [cm/s]**	31.2 ± 1.1	31.4 ± 1.8	30.3 ± 1.3	p > 0.05	31.9 ± 1.0	33.3 ± 1.9	32.1 ± 2.0	p > 0.05
**time spent in the target quadrant [s]**	19.7 ± 3.0	15.2 ± 5.0	16.9 ± 3.1	p > 0.05	26.6 ± 3.6	20.3 ± 5.1	13.2 ± 3.3	p > 0.05
**time spent in quadrant 2 [s]**	19.7 ± 1.8	10.9 ± 0.9	13.7 ± 2.4	p > 0.05	25.5 ± 2.6	10.6 ± 1.5	13.1 ± 2.7	p > 0.05
**time spent in quadrant 3 [s]**	14.3 ± 1.1	7.2 ± 1.7	6.6 ± 1.0	p > 0.05	13.8 ± 1.3	6.4 ± 0.8	8.0 ± 1.3	p > 0.05
**time spent in quadrant 4 [s]**	10.3 ± 3.5	7.7 ± 2.1	6.8 ± 2.1	p > 0.05	17.4 ± 1.8	9.1 ± 2.0	12.6 ± 3.5	p > 0.05

Supplementary Table S6. Comparison at RODA analysis of Morris water maze training between the sham, animals with short and long latency group at weeks 9 and 27 after stimulation (Mean±SD).

RODA analysis of Morris Water Maze Training			Week 9				Week 27			
**Type:**	Day:	Trial:	Sham [%]	Short latency [%]	Long latency [%]	p value	Sham [%]	Short latency [%]	Long latency [%]	p value
**Thigmotaxis**	day 1	1	12.0±16.4	61.6±22.9	52.6±10.7	p > 0.05	4.2±4.7	11.4±20.1	5.8±7.4	p > 0.05
		2	2.0±4.1	30.7±36.6	2.6±6.3	p > 0.05	0±0	5.7±12.7	1.1±2.9	p > 0.05
	day 2	1	25.0±15.2	39.0±35.3	12.5±11.8	p > 0.05	0.3±1.1	14.2±30.0	12.1±20.9	p > 0.05
		2	8.1±10.2	11.8±9.2	7.0±8.9	p > 0.05	0±0	4.2±9.5	0±0	p > 0.05
	day 3	1	13.8±16.9	23.6±35.5	10.4±18.2	p > 0.05	1.3±3.1	24.±48.2	10.9±0	p > 0.05
		2	1.5±3.3	9.3±18.7	1.8±2.7	p > 0.05	3.8±5.3	16.7±27.3	1.7±4.3	p > 0.05
**Incrusion**	day 1	1	7.1±8.4	11.1±7.8	13.3±11.4	p > 0.05	5.2±6.0	9.7±10.9	5.5±9.4	p > 0.05
		2	6.9±6.2	9.3±5.9	6.7±7.2	p > 0.05	2.9±4.4	7.2±5.7	4.7±6.2	p > 0.05
	day 2	1	10.2±4.8	15.1±8.2	18.7±7.3	p > 0.05	3.3±4.6	6.0±7.1	10.1±8.1	p > 0.05
		2	3.7±5.5	15±10.4	0±0	p > 0.05	0±0	5±11.1	5.3±6.8	p > 0.05
	day 3	1	16.0±17.0	4.9±6.7	8.8±10.5	p > 0.05	4.2±7.3	4.2±1.5	7.1±6.8	p > 0.05
		2	4.2±6.8	3.9±7.8	3.1±4.4	p > 0.05	3.5±4.3	5.3±6.8	3.8±6.6	p > 0.05
**Scanning**	day 1	1	19.3±1.5	0.4±1.1	0.4±1.1	p > 0.05	6.9±6.3	7.5±6.4	7.1±4.4	p > 0.05
		2	6.2±5.3	61.5±2.6	7.8±12.4	p > 0.05	4.7±3.6	10.5±13.2	6.2±3.9	p > 0.05
	day 2	1	6.5±6.6	3.6±5.7	11.9±8.0	p > 0.05	9.5±8.6	4.5±8.4	9.1±7.7	p > 0.05
		2	3.1±4.4	5.6±4.0	3.1±3.6	p > 0.05	4.9±1.7	7.8±5.8	7.7±9.4	p > 0.05
	day 3	1	6.2±3.4	7.1±5.6	10.9±8.3	p > 0.05	4.9±5.3	3.3±2.9	11.2±9.4	p > 0.05
		2	10.1±8.6	8.5±6.4	6.8±10.9	p > 0.05	1.9±2.8	6.8±7.7	7.9±7.1	p > 0.05
**Focused search**	day 1	1	22.2±3.1	0.8±2.3	0±0	p > 0.05	2.3±4.3	9.0±8.9	4.5±9.3	p > 0.05
		2	0±0	0.5±1.2	0.5±1.2	p > 0.05	1.4±4.3	2.2±4.2	5.2±6.2	p > 0.05
	day 2	1	2.8±4.0	1.5±3.8	1.5±3.8	p > 0.05	3.9±5.2	0±0	2.3±6.0	p > 0.05
		2	1.8±4.1	3.7±8.3	2.3±4.6	p > 0.05	0.8±1.7	0±0	0±0	p > 0.05
	day 3	1	0±1.1	1.7±4.7	2.0±5.1	p > 0.05	1.0±2.3	0±0	0±0	p > 0.05
		2	2.3±6.6	0±0	4.3±2.9	p > 0.05	0±0	0±0	1.1±2.9	p > 0.05
**Self-orienting**	day 1	1	26.1±0	0.8±0.8	0.8±8.1	p > 0.05	7.6±8.6	11.5±12.4	5.4±6.6	p > 0.05
		2	2.7±4.2	2.6±2.3	3.6±2.3	p > 0.05	3.0±4.8	6.5±4.5	1.1±2.9	p > 0.05
	day 2	1	0.5±1.8	1.5±4.1	2.6±4.1	p > 0.05	7.1±6.5	0.6±1.4	10.8± 13.3	p > 0.05
		2	3.7±8.3	1.8±3.8	10.1±4.1	p > 0.05	0.8±1.6	0±0	1.6±3.2	p > 0.05
	day 3	1	2.2±3.4	0.8±2.7	2.6±6.9	p > 0.05	1.6±2.3	0±0	1.4±3.2	p > 0.05
		2	5.0±6.6	0±0	6.8±7.7	p > 0.05	1.4±3.1	0±0	0±0	p > 0.05
**Scanning surroundings**	day 1	1	32.2±8.0	7.1±8.9	5.8±4.9	p > 0.05	7.0±8.7	4.9±5.9	2.0±4.0	p > 0.05
		2	3.8± 4.0	4.1±6.4	8.3±6.1	p > 0.05	1.4±2.3	5.5±7.4	4.7±6.2	p > 0.05
	day 2	1	4.2±4.2	4.1±3.7	6.2±5.2	p > 0.05	5.5±8.6	12.3±19.1	4.3±4.3	p > 0.05
		2	5±4.7	6.8±10.9	4.6±5.4	p > 0.05	5.9±4.4	4.0±5.5	0±0	p > 0.05
	day 3	1	4.4±5.9	2.3±5.9	4.1±6.7	p > 0.05	3.3±3.9	0.8±1.6	9.1±0	p > 0.05
		2	1.1±2.3	4.6±7.4	2.5±5.5	p > 0.05	3.4±4.9	4.8±5.5	7.5±13.1	p > 0.05
**Scanning target**	day 1	1	40.3±2.7	0±0	1.3±3.5	p > 0.05	4.1±7.6	0±0	1.3±3.6	p > 0.05
		2	5±9.0	4.6±8.2	4.6±8.2	p > 0.05	6.6±8.5	3.8±8.6	2.8±7.5	p > 0.05
	day 2	1	1.6±5.7	3.7±7.5	2.8±7.5	p > 0.05	8.3±9.3	2.6±3.5	2.4±4.3	p > 0.05
		2	14.2±8.7	7.5±9.6	10.3±10.0	p > 0.05	12.7±10.0	0±0	5.9±10.0	p > 0.05
	day 3	1	9.3±9.6	0±0	2.8±7.5	p > 0.05	1.8±6.0	5.9±8.9	4.7±10.0	p > 0.05
		2	6.6±9.8	8.5±10.6	6.6±9.4	p > 0.05	11.6±10.2	5.2±8.6	6.0±7.2	p > 0.05

Supplementary Figure S7 Comparison of RODA analysis of the Morris water maze test day between the sham, short-latency and long-latency groups at weeks 9 and 27 after stimulation.

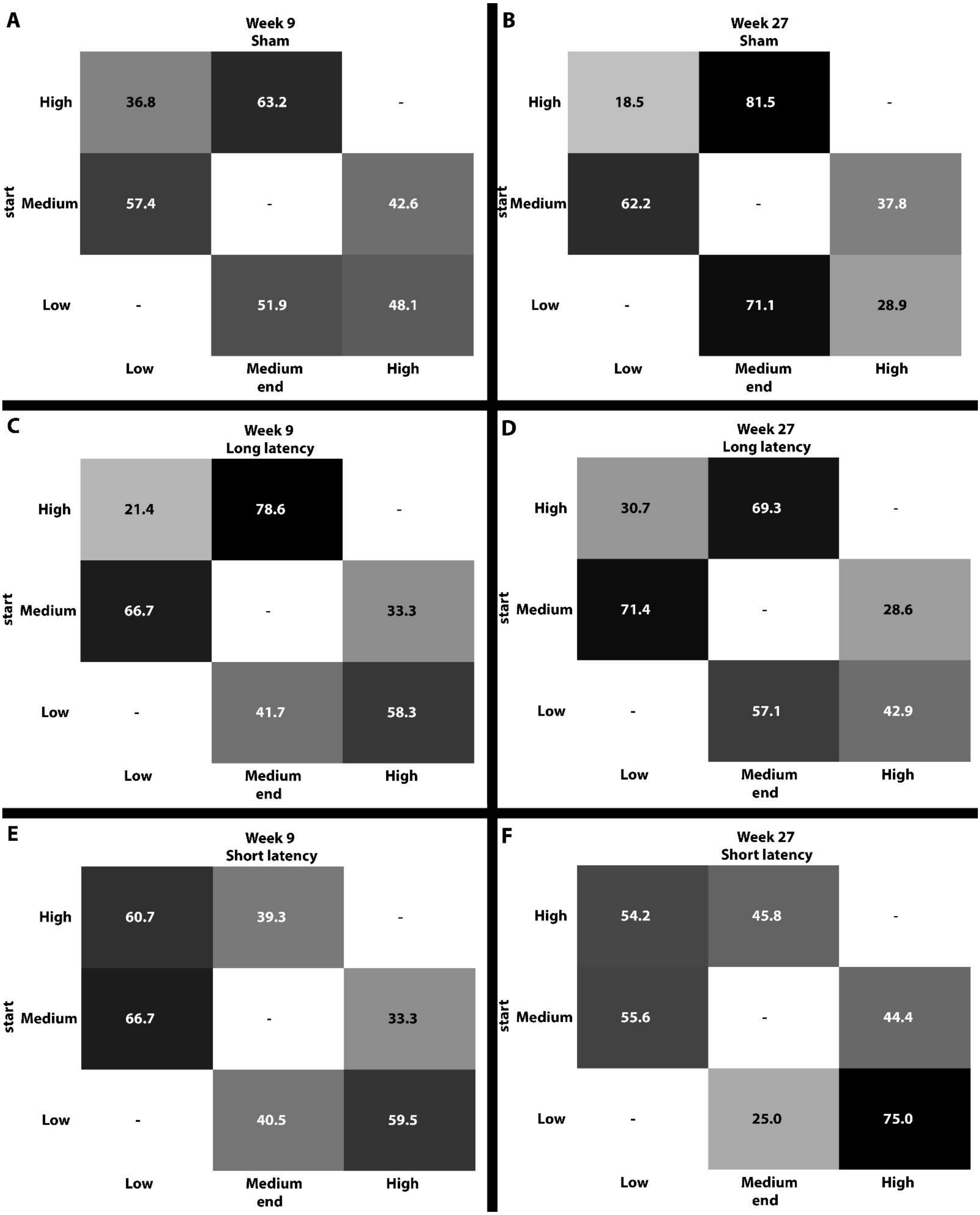

The probabilities (%) of transitioning between strategies were checked in test trials (weeks 9 and 27) in the sham, long-latency, and short-latency groups. Rows and columns indicate the starting and ending strategies, respectively. Row values (for the same starting strategy) were normalized (sum of each row equals 100%). Low strategies: thigmotaxis, incursion. Medium strategies: scanning, focused search, chaining reaction, self-orienting. High strategies: scanning surrounding, scanning target. Thigmotaxis and incursion were assigned to low-level strategies

because the animals mostly stayed in areas close to the walls of the arena. Scanning, focused search, chaining response, and self-orienting were assigned to medium-level strategies because the animals explored inner parts of the arena. Scanning surroundings and scanning target were assigned to high-level strategies because the animals passed or focused on areas of the arena that contained the platform. Sham animals at week 9 transitioned more often from medium- to high-level strategies than the other two groups; similar conclusion is obtained for week 27 but only between sham and long latency groups. The sham and long-latency groups at both weeks 9 and 27 transitioned more often from high- to medium-level strategies. The short-latency group at week 9 transitioned much more often from high- to low-level strategies and during week 27 the difference on the transition probabilities between high and low and high and medium level strategies are more equalized. For the transition probabilities between low and medium and low and high strategies transitions the sham groups increases the transitions of the former between weeks 9 and 27 while the short latency group does the opposite, it starts from more equalized transitions between low and medium and low and high strategies on week 9 but on week 27 it increases the latter. The long-latency group did not exhibit such differences between weeks 9 and 27, and the transitions from low-level strategies to medium- and high-level strategies were more equalized.

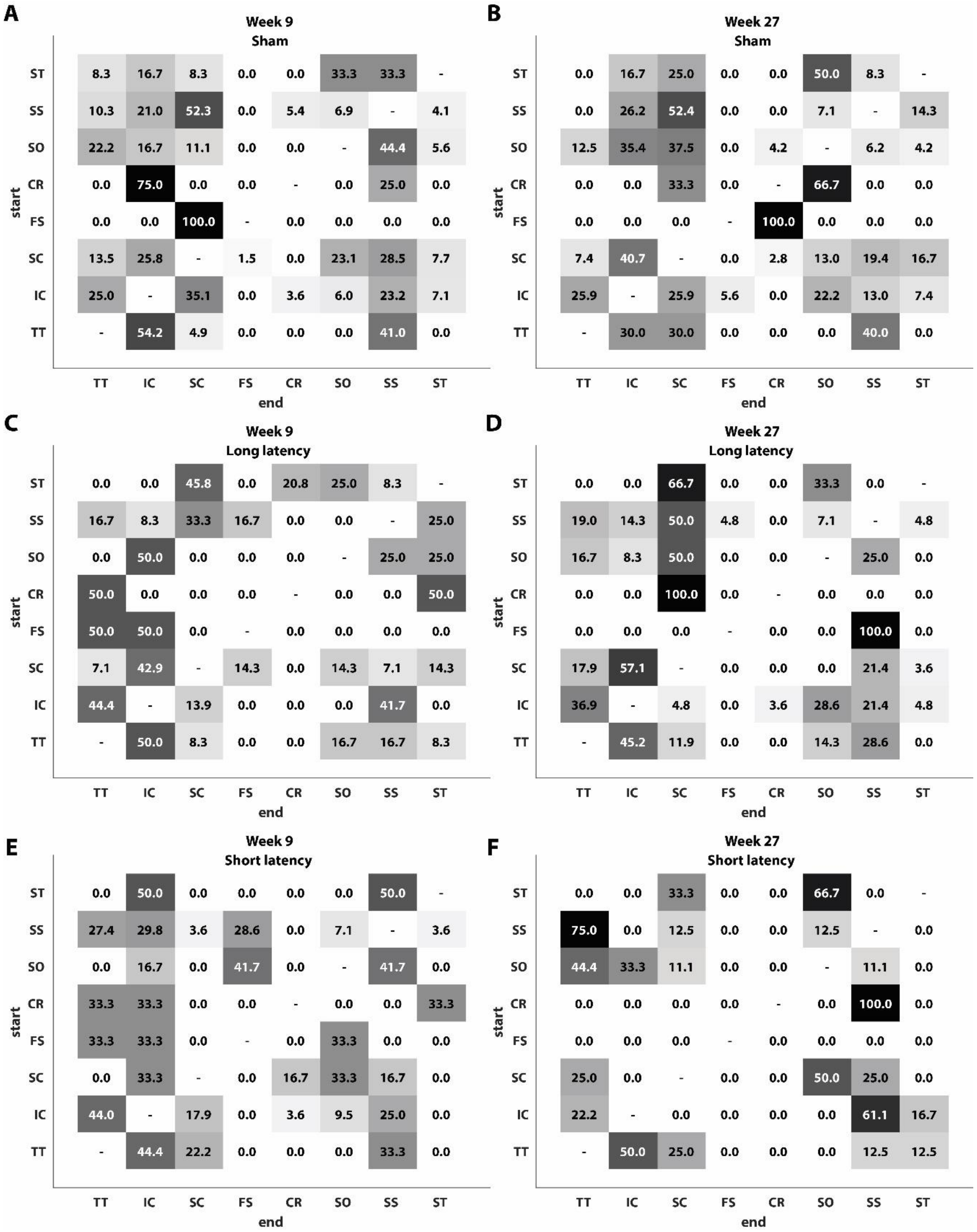

Supplementary Table S8. Behavioral changes between the sham, non-epileptic and epileptic animals (Mean±SD) .

A

Behavioral hyperexcitability test	Week 6<br>[median (range: min ; max)]			
**Type:**	Sham	Non-epileptic	Epileptic	p value
**approach response**	2 (1;4)	2 (1;4)	2 (1;2)	p > 0.05
**touch response**	2 (1;3)	2 (1;4)	1,5 (1;5)	p > 0.05
**loud noise**	2 (1;3)	2 (1;3)	2 (1;3)	p > 0.05
**pick-up**	1 (1;5)	1 (1;5)	1,5 (1;5)	p > 0.05

B

Open field test	Week 8			
**Type:**	Sham	Non-epileptic	Epileptic	p value
**latency to enter inner area of the arena [s]**	40 ± 24.7	19.4 ± 11.6	30.8 ± 16.1	p > 0.05
**latency to enter the central area [s]**	142.8 ± 36.6	184.1 ± 87.8	77.9 ± 77.9	p > 0.05
**speed [cm/s]**	4.6 ± 0.4	5.2 ± 0.5	6.0 ± 0.7	p > 0.05

C

Novel object exploration test	Week 8			
**Type:**	Sham	Non-epileptic	Epileptic	p value
**latency to enter the inner area of the arena [s]**	59.0 ± 48.3	13.3 ± 4.2	36.4 ± 22.6	p > 0.05
**latency to approach the novel object [s]**	63.6 ± 48.3	19.9 ± 4.9	44.1 ± 26.2	p > 0.05
**mobility [s]**	54.4 ± 18.4	38.5 ± 20.8	134.9 ± 68.1	p > 0.05

D

Elevated plus maze test	Week 8			
**Type:**	Sham	Non-epileptic	Epileptic	p value
**number of entries into the closed arms**	17.7 ± 1.7	17.2 ± 2.7	15.1 ± 2.0	p > 0.05
**number of entries into the open arms**	10.6 ± 2.2	4.8 ± 2.2	9.7 ± 3.7	p > 0.05
**speed [cm/s]**	6.1 ± 0.3	5.6 ± 0.6	6.5 ± 1.0	p > 0.05

E

Morris water maze test	Week 9			
**Type:**	Sham	Non-epileptic	Epileptic	p value
**swimming time over the platform [s]**	1.9 ± 0.2	0.8 ± 0.1	1.4 ± 0.5	p > 0.05
**speed [cm/s]**	31.2 ± 1.1	30.2 ± 1.4	31.5 ± 1.8	p > 0.05
**time spent in the target quadrant [s]**	19.7 ± 3.0	24.8 ± 5.5	27.4 ± 5.8	p > 0.05
**time spent in quadrant 2 [s]**	19.7 ± 1.8	22.7 ± 3.8	16.8 ± 22.7	p > 0.05
**time spent in quadrant 3 [s]**	14.3 ± 1.1	11.7 ± 1.0	10.6 ± 3.1	p > 0.05
**time spent in quadrant 4 [s]**	10.3 ± 3.5	8.4 ± 2.8	15.8 ± 3.6	p > 0.05

Supplementary Table S9. Comparison at RODA analysis of Morris water maze training between the sham, non-epileptic and epileptic animals at week 9 after stimulation (Mean±SD).

**RODA analysis of Morris Water Maze Training**			**9 week**			
**Type:**	Day:	Trial:	Sham [%]	Non-epileptic [%]	Epileptic [%]	p value
**Thigmotaxis**	day 1	1	12.0±16.4	54.6±7.2	59.8±24.7	p > 0.05
		2	2.0±4.1	2.6±6.3	36.8± 37.3	p > 0.05
	day 2	1	25.0±15.2	9.8±11.0	42.7±31.4	p > 0.05
		2	8.1±10.2	3.7±8.3	10.7±7.8	**p < 0.05**
	day 3	1	13.8±16.9	5.6±5.5	30.3±34.7	p > 0.05
		2	1.5±3.3	2.0±3.6	8.1±16.4	p > 0.05
**Incrusion**	day 1	1	7.1±8.4	11.7±8.7	12.9±10.1	p > 0.05
		2	6.9±6.2	6.7±7.2	11.2±4.1	p > 0.05
	day 2	1	10.2±4.8	19.1±10.7	11.4±2.5	p > 0.05
		2	3.7±5.5	1.2±2.7	11.6±10.4	p > 0.05
	day 3	1	16.0±17.0	4.2±10.6	6.6±6.8	p > 0.05
		2	4.2±6.8	4.1±3.6	5.0±7.1	p > 0.05
**Scanning**	day 1	1	19.3±1.5	0±0	0.8±1.5	p > 0.05
		2	6.2±5.3	7.8±12.4	1.8±2.7	p > 0.05
	day 2	1	6.5±6.6	12.9±5.5	2.0±5.1	p > 0.05
		2	3.1±4.4	5±8.1	4.9±3.9	p > 0.05
	day 3	1	6.2±3.4	13.1±7.7	6.2±3.9	p > 0.05
		2	10.1±8.6	6.2±10.8	11.8±9.2	p > 0.05
**Focused search**	day 1	1	22.2±3.1	1.1±3.3	0.8±2.3	p > 0.05
		2	0±0	0.5±1.2	0±0	p > 0.05
	day 2	1	2.8±4.0	2.6±4.5	0±0	p > 0.05
		2	1.8±4.1	0±0	4.0±7.3	p > 0.05
	day 3	1	0±1.1	5±6.8	0±0	p > 0.05
		2	2.3±6.6	1.0±1.8	3.7±8.3	p > 0.05
**Self-orienting**	day 1	1	26.1±0	0.7±2.2	0.8±2.3	p > 0.05
		2	2.7±4.2	3.6±4.1	1.8±4.1	p > 0.05
	day 2	1	0.5±1.8	3.5±4.5	0±0	p > 0.05
		2	3.7±8.3	5.6±4.6	4.0±6.9	p > 0.05
	day 3	1	2.2±3.4	1.2±2.7	2.2±3.9	p > 0.05
		2	5.0±6.6	3.1±5.4	3.7±8.3	p > 0.05
**Scanning surroundings**	day 1	1	32.±8.0	5.8±5.1	6.2±9.0	p > 0.05
		2	3.8 ± 4.0	8.3±6.1	5±6.8	p > 0.05
	day 2	1	4.2±4.2	4.0±5.3	5.7±3.6	p > 0.05
		2	5±4.7	3.1±4.4	7.1±8.9	p > 0.05
	day 3	1	4.4±5.9	3.1±6.9	3.5±6.3	p > 0.05
		2	1.1±2.3	6.2±6.2	3.7±6.7	p > 0.05
**Scanning target**	day 1	1	40.3±2.7	0±0	1.3±3.5	p > 0.05
		2	5±9.0	6.5±9.3	7.5±9.6	p > 0.05
	day 2	1	1.6±5.7	2.5±7.0	3.7±7.5	p > 0.05
		2	14.2±8.7	9.0±9.9	1.7±4.7	p > 0.05
	day 3	1	9.3±9.6	3.3±8.1	0±0	p > 0.05
		2	6.6±9.8	11.0±0.0	5.7±9.7	p > 0.05

Supplementary Figure S10 Comparison of RODA analysis of Morris water maze test day between the sham, non-epileptic and epileptic animals at week 9 after stimulation.

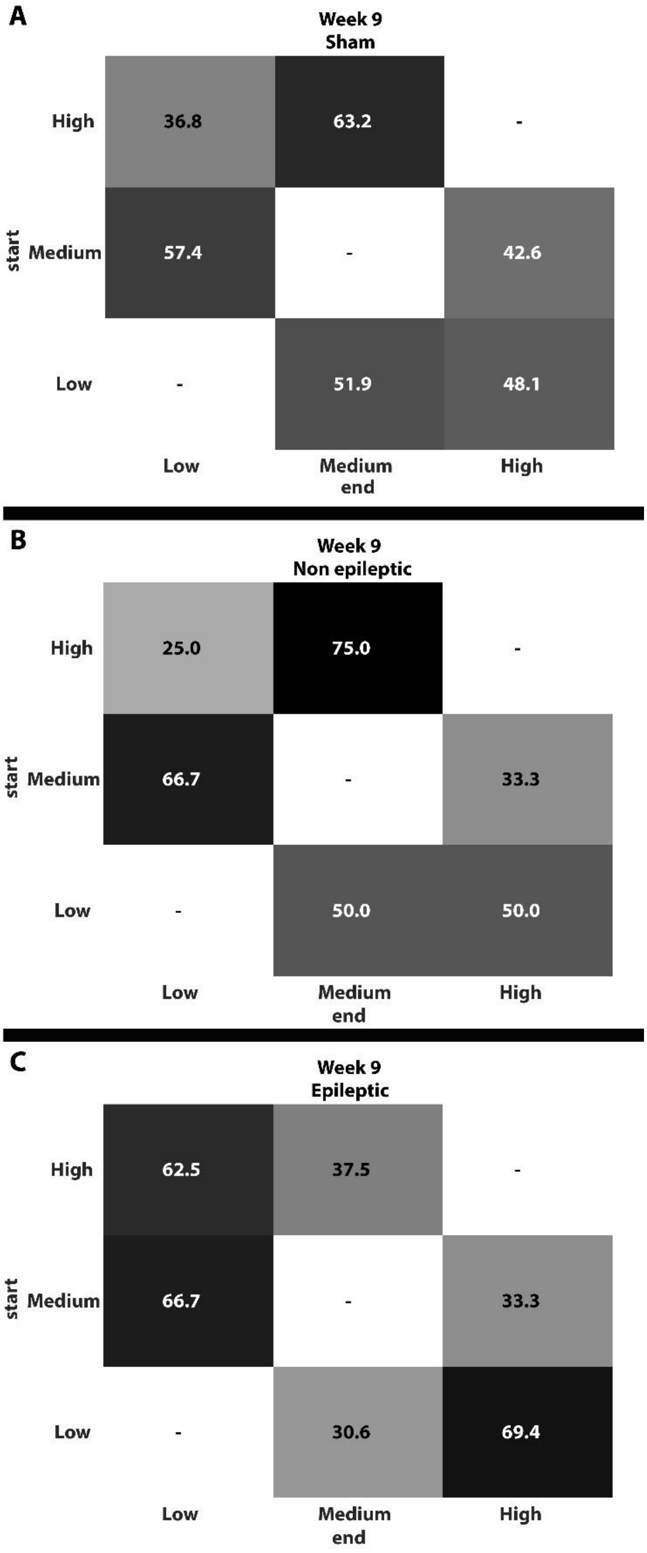

The probabilities (%) of transitioning between strategies were checked in test trials at week 9 in the sham, non-epileptic, and epileptic animals. Rows and columns indicate the starting and ending strategies, respectively. Row values (for the same starting strategy) were normalized (sum of each row equals 100%). TT, thigmotaxis; IC, incursion; SC, scanning; FS, focused search; CR, chaining reaction; SO, self-orienting; SS, scanning surrounding; ST, scanning target. Thigmotaxis and incursion were assigned to low-level strategies because the animals mostly stayed in areas close to the walls of the arena. Scanning, focused search, chaining response, and self-orienting were assigned to intermediate-level strategies because the animals explored inner parts of the arena. Scanning surroundings and scanning target were assigned to high-level strategies because the animals passed or focused on areas of the arena that

contained the platform. Epileptic animals had a higher probability of transitioning between low- and high-level strategies. The sham and non-epileptic groups had nearly equal probabilities of transitioning between low- and medium-level strategies and between low- and high-level strategies. Sham animals had more equalized probabilities of transitioning between medium- and low-level strategies and medium- and high-level strategies. The non-epileptic and epileptic groups had a higher probability of transitioning between medium- and low-level strategies. The sham and non-epileptic groups had a higher probability of transitioning between high- and medium-level strategies than the epileptic group, which had a higher probability of transitioning between high- and low-level strategies.

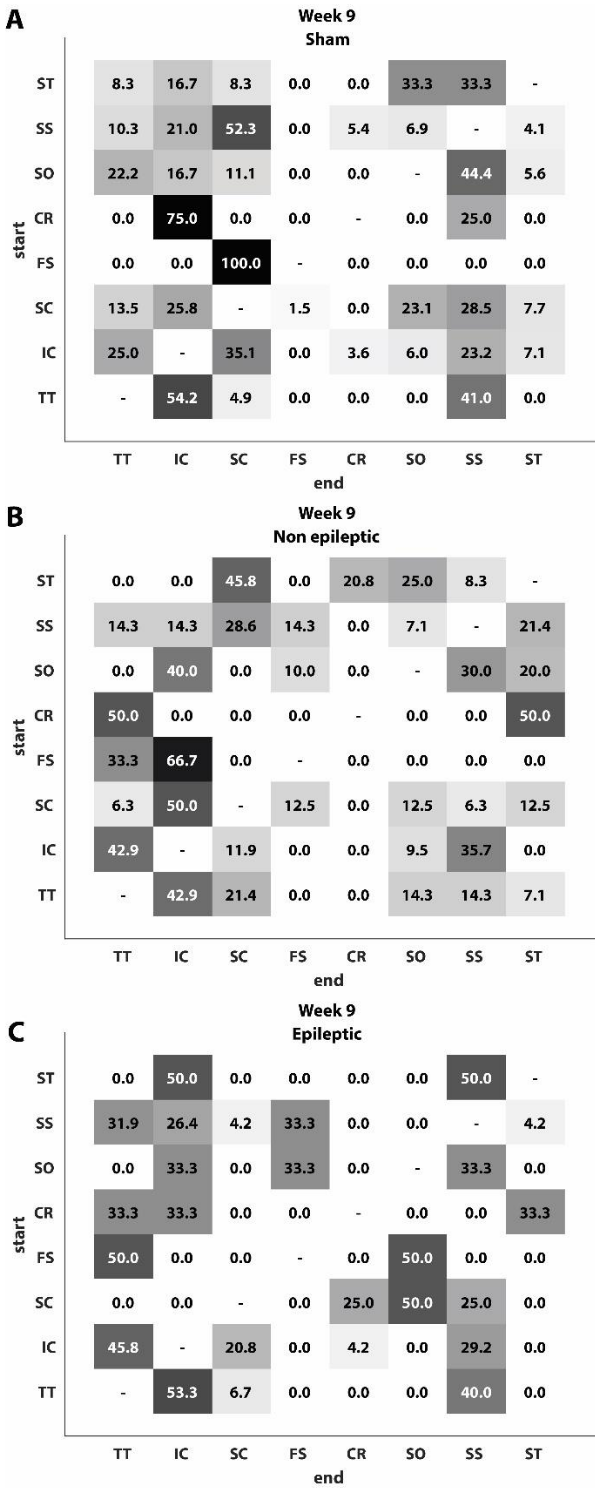

Supplementary Table S11. Behavioral changes between the sham, low seizure number and high seizure number groups (Mean±SD).

A

Behavioral hyperexcitability test	Week 6 [median (range: min ; max)]				Week 12 [median (range: min ; max)]			
**Type:**	Sham	Low seizure numer	High seizure numer	p value	Sham	Low seizure numer	High seizure numer	p value
**approach response**	2 (1;4)	2 (1;4)	2 (1;3)	p > 0.05	2 (1;4)	2 (1;4)	2 (1;3)	p > 0.05
**touch response**	2 (1;3)	2 (1;3)	2 (1;5)	p > 0.05	2 (1;4)	3 (1;5)	2,5 (1;5)	p > 0.05
**loud noise**	2 (1;3)	2 (1;3)	2 (1;2)	p > 0.05	2 (1;3)	2 (1;3)	2 (1;2)	p > 0.05
**pick-up**	1 (1;5)	1 (1;5)	1 (1;5)	p > 0.05	1 (1;5)	1 (1;5)	1 (1;5)	p > 0.05

B

Open field test	Week 8				Week 26			
**Type:**	Sham	Low seizure numer	High seizure numer	p value	Sham	Low seizure numer	High seizure numer	p value
**latency to enter inner area of the arena [s]**	40 ± 24.7	21.6 ± 12.8	26.9 ± 14.5	p > 0.05	119.4 ± 108.7	65.9 ± 40.3	48.9 ± 35.0	p > 0.05
**latency to enter the central area [s]**	142.8 ± 36.6	212.4 ± 104.7	64.8 ± 52.7	p > 0.05	394.7 ± 131.8	141.8 ± 57.4	141.3 ± 59.1	p > 0.05
**speed [cm/s]**	4.6 ± 0.4	5.3 ± 0.7	5.8 ± 0.5	p > 0.05	4.4 ± 0.4	4.8 ± 0.5	5.1 ± 0.7	p > 0.05

C

Novel object exploration test	Week 8				Week 26			
**Type:**	Sham	Low seizure numer	High seizure numer	p value	Sham	Low seizure numer	High seizure numer	p value
**latency to enter the inner area of the arena [s]**	59.0 ± 48.3	36.2 ± 18.6	8.9 ± 5.2	p > 0.05	11.2 ± 1.7	10.6 ± 4.8	16.5 ± 14.6	p > 0.05
**latency to approach the novel object [s]**	63.6 ± 48.3	41.9 ± 17.7	11.6 ± 5.6	p > 0.05	12.6 ± 1.8	12.6 ± 1.8	15.2 ± 6.4	p > 0.05
**mobility [s]**	54.4 ± 18.4	70.3 ± 49.6	108.6 ± 63.2	p > 0.05	133.9 ± 23.8	202.3 ± 25.3	190.3 ± 48.7	p > 0.05

D

Elevated plus maze test	Week 8				Week 26			
**Type:**	Sham	Low seizure numer	High seizure numer	p value	Sham	Low seizure numer	High seizure numer	p value
**number of entries into the closed arms**	17.7 ± 1.7	17.2 ± 2.0	22.2 ± 3.1	p > 0.05	22.2 ± 3.0	19.7 ± 3.3	35.0 ± 5.1	**p < 0.05**
**number of entries into the open arms**	10.6 ± 2.2	6.5 ± 2.5	7.8 ± 3.6	p > 0.05	3.0 ± 0.9	3.8 ± 2.7	3.1 ± 2.5	p > 0.05
**speed [cm/s]**	6.1 ± 0.3	6.0 ± 0.7	6.1 ± 0.9	p > 0.05	4.1 ± 0.2	4.2 ± 0.2	4.5 ± 0.4	p > 0.05

E

Morris water maze test	Week 9				Week 27			
**Type:**	Sham	Low seizure numer	High seizure numer	p value	Sham	Low seizure numer	High seizure numer	p value
**swimming time over the platform [s]**	1.9 ± 0.2	1.6 ± 0.2	1.9 ± 0.5	p > 0.05	2.3 ± 0.2	0.9 ± 0.2	2.7 ± 0.5	p > 0.05
**speed [cm/s]**	31.2 ± 1.1	30.4 ± 1.2	31.4 ± 2.1	p > 0.05	31.9 ± 1.0	32.8 ± 2.7	32.5 ± 1.4	p > 0.05
**time spent in the target quadrant [s]**	19.7 ± 3.0	25.0 ± 6.7	26.7 ± 5.0	p > 0.05	26.6 ± 3.6	23.5 ± 6.2	28.6 ± 7.3	p > 0.05
**time spent in quadrant 2 [s]**	19.7 ± 1.8	22.0 ± 5.5	18.6 ± 1.1	p > 0.05	25.5 ± 2.6	22.4 ± 4.7	17.7 ± 3.6	p > 0.05
**time spent in quadrant 3 [s]**	14.3 ± 1.1	11.4 ± 1.8	11.0 ± 2.3	p > 0.05	13.8 ± 1.3	11.1 ± 1.1	12.5 ± 2.4	p > 0.05
**time spent in quadrant 4 [s]**	10.3 ± 3.5	8.2 ± 3.5	14.1 ± 3.0	p > 0.05	17.4 ± 1.8	21.6 ± 7.3	14.6 ± 2.4	p > 0.05

Supplementary Table S12. Comparison at RODA analysis of Morris water maze training between the sham, low seizure number and high seizure number groups at weeks 9 and 27 after stimulation (Mean±SD).

RODA analysis of Morris Water Maze Training			Week 9				Week 27			
**Type:**	Day:	Trial:	Sham [%]	Low seizure numer [%]	High seizure numer [%]	p value	Sham [%]	Low seizure numer [%]	High seizure numer [%]	p value
**Thigmotaxis**	day 1	1	12.0±16.4	64.28±17.9	50.0±36.7	p > 0.05	4.2±4.7	6.6±17.5	9.3±6.7	p > 0.05
		2	2.0±4.1	23.21±36.8	7.5±10.5	p > 0.05	0±0	4.7±11.6	2.3±4.1	p > 0.05
	day 2	1	25.0±15.2	21.25±24.9	29.0±32.7	p > 0.05	0.3±1.1	13.4±26.9	4.3±6.7	p > 0.05
		2	8.1±10.2	13.75±9.52	5.0±4.7	p > 0.05	0±0	7.1±12.3	0±0	p > 0.05
	day 3	1	13.8±16.9	16.14±28.0	18.7±31.3	p > 0.05	1.3±3.1	22.5±41.5	2.6±5.3	p > 0.05
		2	1.5±3.3	0±0	9.3±15.9	p > 0.05	3.8±5.3	13.3±24.8	0±0	p > 0.05
**Incrusion**	day 1	1	7.1±8.4	8.92±6.85	15.6±10.9	p > 0.05	5.2±6.0	7.9±9.8	8.0±11.8	p > 0.05
		2	6.9±6.2	8.92±6.61	6.8±6.7	p > 0.05	2.9±4.4	5.4±6.0	6.9±7.1	p > 0.05
	day 2	1	10.2±4.8	17.5±7.84	16.5±8.2	p > 0.05	3.3±4.6	8.4±6.3	3.3±3.9	p > 0.05
		2	3.7±5.5	10±10.45	6.2±10.8	p > 0.05	0±0	8.3±14.4	1.7±3.5	p > 0.05
	day 3	1	16.0±17.0	8.8±10.53	4.9±6.7	p > 0.05	4.2±7.3	5.8±4.4	3.4±2.9	p > 0.05
		2	4.2±6.8	0±0	6.2±6.6	p > 0.05	3.5±4.3	6.0±8.3	4.5±3.9	p > 0.05
**Scanning**	day 1	1	19.3±1.5	0±0	0.8±1.5	p > 0.05	6.9±6.3	6.1±3.9	11.1±4.8	p > 0.05
		2	6.2±5.3	3.57±7.08	6.2±12.3	p > 0.05	4.7±3.6	11.6±11.3	3.4±3.5	p > 0.05
	day 2	1	6.5±6.6	9.37±10.36	6.6±6.3	p > 0.05	9.5±8.6	6.0±7.8	10.1±9.9	p > 0.05
		2	3.1±4.4	3.75±4.07	4.3±4.1	p > 0.05	4.9±1.7	8.6±8.1	9.5±8.0	p > 0.05
	day 3	1	6.2±3.4	10.4±9.4	7.5±4.3	p > 0.05	4.9±5.3	7.3±8.9	9.4±6.1	p > 0.05
		2	10.1±8.6	8.59±6.44	6.8±10.9	p > 0.05	1.9±2.8	2.0±3.1	8.±9.0	p > 0.05
**Focused search**	day 1	1	22.2±3.1	0±0	0.8±2.3	p > 0.05	2.3±4.3	4.1±7.6	5.8±7.6	p > 0.05
		2	0±0	0.44±1.18	0.6±1.3	p > 0.05	1.4±4.3	4.4±5.0	5.8±7.4	p > 0.05
	day 2	1	2.8±4.0	1.875±4.19	1.3±3.5	p > 0.05	3.9±5.2	2.6±6.5	0±0	p > 0.05
		2	1.8±4.1	3.75±8.38	1.8±4.1	p > 0.05	0.8±1.7	0±0	0±0	p > 0.05
	day 3	1	0±1.1	2.0±5.1	1.7±4.7	p > 0.05	1.0±2.3	1.9±4.3	1.7±3.5	p > 0.05
		2	2.3±6.6	0.78±1.56	3.7±8.3	p > 0.05	0±0	0±0	0±0	p > 0.05
**Self-orienting**	day 1	1	26.1±0	0.89±2.36	0.8±2.3	p > 0.05	7.6±8.6	3.7±4.7	12.7±13.5	p > 0.05
		2	2.7±4.2	2.32±3.91	4.3±4.1	p > 0.05	3.0±4.8	3.2±5.3	6.9±0.3	p > 0.05
	day 2	1	0.5±1.8	1.87±4.19	2.2±3.9	p > 0.05	7.1±6.5	5.0±4.6	12.2±18.5	p > 0.05
		2	3.7±8.3	3.125±5.41	6.8±7.1	p > 0.05	0.8±1.6	0±0	1.6±3.2	p > 0.05
	day 3	1	2.2±3.4	2.08±3.22	1.3±3.5	p > 0.05	1.6±2.3	0±0	0±0	p > 0.05
		2	5.0±6.6	2.34±4.68	5±8.1	p > 0.05	1.4±3.1	0±0	0±0	p > 0.05
**Scanning surroundings**	day 1	1	32.2±8.0	6.69±4.91	6.2±9.0	p > 0.05	7.0±8.7	0.9±2.4	7.0±6.5	p > 0.05
		2	3.8± 4.0	5.8±7.3	6.8±5.5	p > 0.05	1.4±2.3	6.3±6.9	4.7±8.2	p > 0.05
	day 2	1	4.2±4.2	3.125±3.12	6.6±4.9	p > 0.05	5.5±8.6	10.8±17.6	3.4±5.0	p > 0.05
		2	5±4.7	3.75±5.13	8.1±10.2	p > 0.05	5.9±4.4	6.7 ± 5.9	0±0	p > 0.05
	day 3	1	4.4±5.9	0±0	5.8±7.5	p > 0.05	3.3±3.9	4.5±10.0	8.8±15.6	p > 0.05
		2	1.1±2.3	0.78±1.56	5.6±7.7	p > 0.05	3.4±4.9	1.9±4.3	4.3±4.9	p > 0.05
**Scanning target**	day 1	1	40.3±2.7	0±0	1.3±3.5	p > 0.05	4.1±7.6	1.3±3.6	0±0	p > 0.05
		2	5±9.0	1.78±4.72	7.5±9.6	p > 0.05	6.6±8.5	0±0	9.8±11.3	p > 0.05
	day 2	1	1.6±5.7	6.6±9.42	0±0	p > 0.05	8.3±9.3	3.9±4.6	1.6±3.2	p > 0.05
		2	14.2±8.7	7.5±9.68	9.3±9.6	p > 0.05	12.7±10.0	10±10.9	1.6±3.2	p > 0.05
	day 3	1	9.3±9.6	2.85±7.55	0±0	p > 0.05	1.8±6.0	4.4±8.0	2.4±4.8	p > 0.05
		2	6.6±9.8	8.57±10.69	6.6±9.4	p > 0.05	11.6±10.2	8.9±8.3	6.6±9.4	p > 0.05

Supplementary Figure S13 Comparison of RODA analysis of Morris water maze test day between the sham, low seizure number and high seizure number groups at weeks 9 and 27 after stimulation

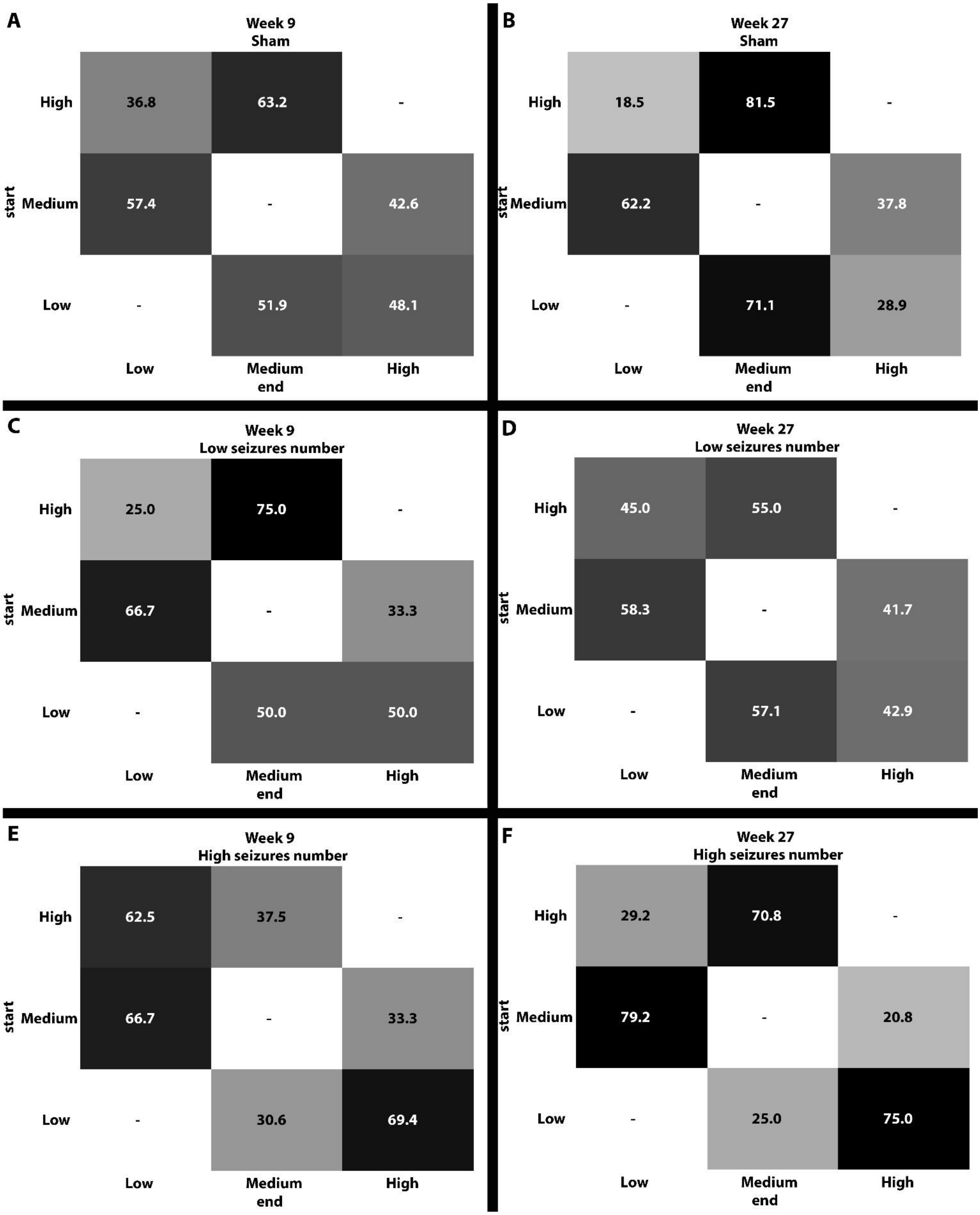

The probabilities (%) of transitioning between strategies were checked in test trials (weeks 9 and 27) in the sham, low-seizure-number, and high-seizure-number groups. Rows and columns indicate the starting and ending strategies, respectively. Row values (for the same starting strategy) were normalized (sum of each row equals 100%). TT, thigmotaxis; IC, incursion; SC, scanning; FS, focused search; CR, chaining reaction; SO, self-orienting; SS, scanning surrounding; ST, scanning target. Thigmotaxis and incursion were assigned to low-level strategies because

the animals mostly stayed in areas close to the walls of the arena. Scanning, focused search, chaining response, and self-orienting were assigned to medium-level strategies because the animals were exploring inner parts of the arena. Scanning surroundings and scanning target were assigned to high-level strategies because the animals passed or focused on areas of the arena that contained the platform. Comparisons of the sham and low-seizure groups at week 9 showed some similarity in the probabilities of transitions between medium- to low-level strategies and high- to medium-level strategies, which were greater than transitions from medium- to high-level strategies and high- to low-level strategies. These differences in the low-seizure group were greater than in the sham group. Both groups had similar equalization between transitions from low- to medium-level strategies and low- to high-level strategies. At week 27, the low-seizure group had more equal probabilities throughout all of the transitions among low-, medium-, and high-level strategies. In the sham group, the probabilities of transitions that started with low-, medium-, and high-level strategies and ended with medium-, low-, and medium-level strategies were higher. The high-seizure group at week 9 had a higher probability of transitioning from a high-level strategy to a low-level strategy compared with the probability of transitioning from a high-level strategy to a low-level strategy. In the other two groups, the opposite was true. Transitions from medium- to low-level strategies were equivalent in both the high- and low-seizure groups, and the probability of transitioning from medium- to low-level strategies was higher than the probability of transitioning from medium- to high-level strategies. In the sham group, these probabilities were more equalized. The probabilities of transitioning from low- to high-level strategies were higher than the probabilities of transitioning from low- to medium-level strategies in the high-seizure group. In the other two groups, the same probabilities were more equalized. At week 27, the high-seizure group had higher probabilities of transitioning from low- to high-levels strategies, from medium- to low-level strategies, and from high- to medium-level strategies.

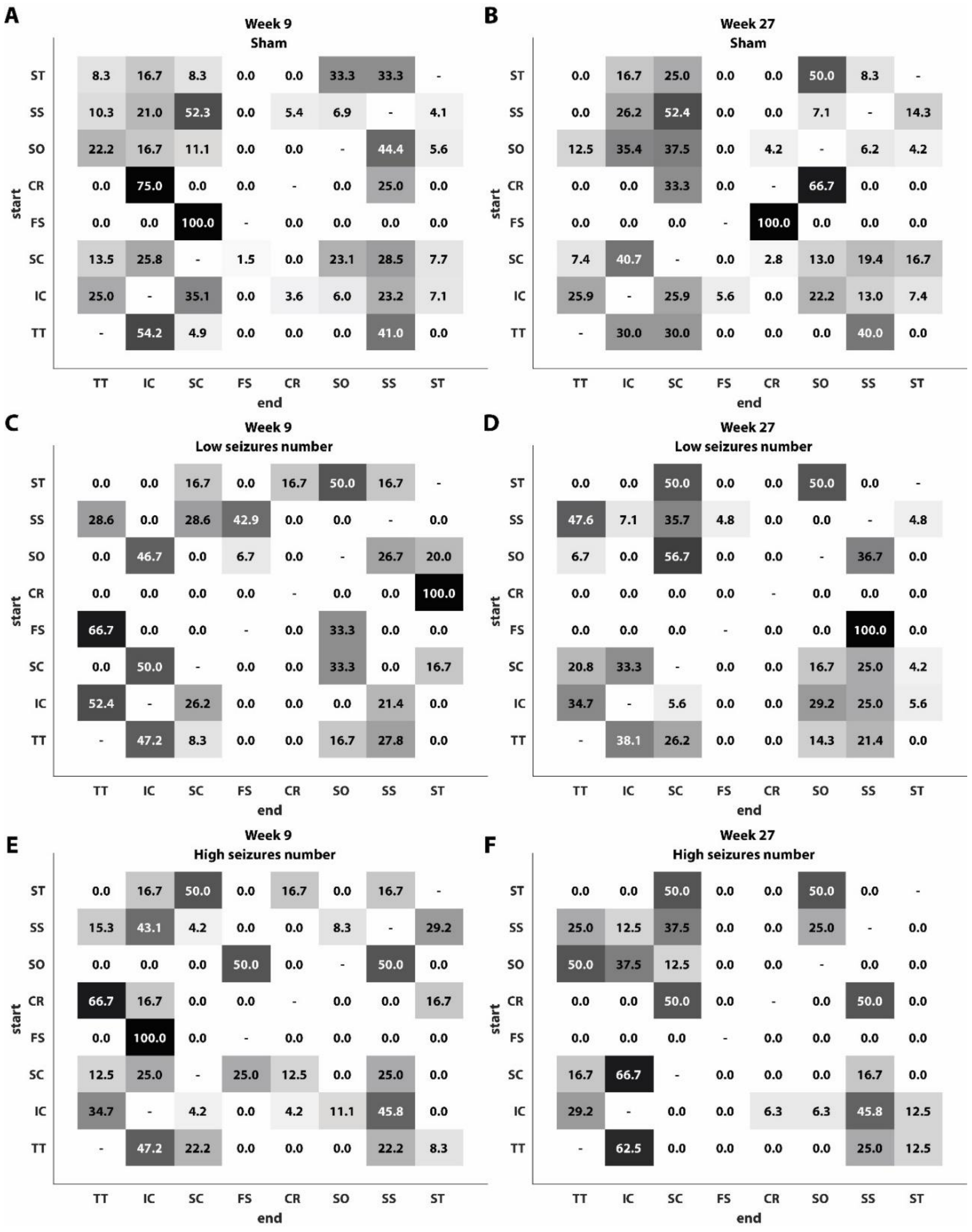
A
Week 9
Sham
ST 8.3 16.7 8.3 0.0 0.0 33.3 33.3 -
SS 10.3 21.0 52.3 0.0 5.4 6.9 - 4.1
SO 22.2 16.7 11.1 0.0 0.0 - 44.4 5.6
CR 0.0 75.0 0.0 0.0 - 0.0 25.0 0.0
FS 0.0 0.0 100.0 - 0.0 0.0 0.0 0.0
SC 13.5 25.8 - 1.5 0.0 23.1 28.5 7.7
IC 25.0 - 35.1 0.0 3.6 6.0 23.2 7.1
TT - 54.2 4.9 0.0 0.0 0.0 41.0 0.0
start
TT IC SC FS CR SO SS ST
end
B
Week 27
Sham
ST 0.0 16.7 25.0 0.0 0.0 50.0 8.3 -
SS 0.0 26.2 52.4 0.0 0.0 7.1 - 14.3
SO 12.5 35.4 37.5 0.0 4.2 - 6.2 4.2
CR 0.0 0.0 33.3 0.0 - 66.7 0.0 0.0
FS 0.0 0.0 0.0 - 100.0 0.0 0.0 0.0
SC 7.4 40.7 - 0.0 2.8 13.0 19.4 16.7
IC 25.9 - 25.9 5.6 0.0 22.2 13.0 7.4
TT - 30.0 30.0 0.0 0.0 0.0 40.0 0.0
start
TT IC SC FS CR SO SS ST
end
C
Week 9
Low seizures number
ST 0.0 0.0 16.7 0.0 16.7 50.0 16.7 -
SS 28.6 0.0 28.6 42.9 0.0 0.0 - 0.0
SO 0.0 46.7 0.0 6.7 0.0 - 26.7 20.0
CR 0.0 0.0 0.0 0.0 - 0.0 0.0 100.0
FS 66.7 0.0 0.0 - 0.0 33.3 0.0 0.0
SC 0.0 50.0 - 0.0 0.0 33.3 0.0 16.7
IC 52.4 - 26.2 0.0 0.0 0.0 21.4 0.0
TT - 47.2 8.3 0.0 0.0 16.7 27.8 0.0
start
TT IC SC FS CR SO SS ST
end
D
Week 27
Low seizures number
ST 0.0 0.0 50.0 0.0 0.0 50.0 0.0 -
SS 47.6 7.1 35.7 4.8 0.0 0.0 - 4.8
SO 6.7 0.0 56.7 0.0 0.0 - 36.7 0.0
CR 0.0 0.0 0.0 0.0 - 0.0 0.0 0.0
FS 0.0 0.0 0.0 - 0.0 0.0 100.0 0.0
SC 20.8 33.3 - 0.0 0.0 16.7 25.0 4.2
IC 34.7 - 5.6 0.0 0.0 29.2 25.0 5.6
TT - 38.1 26.2 0.0 0.0 14.3 21.4 0.0
start
TT IC SC FS CR SO SS ST
end
E
Week 9
High seizures number
ST 0.0 16.7 50.0 0.0 16.7 0.0 16.7 -
SS 15.3 43.1 4.2 0.0 0.0 8.3 - 29.2
SO 0.0 0.0 0.0 50.0 0.0 - 50.0 0.0
CR 66.7 16.7 0.0 0.0 - 0.0 0.0 16.7
FS 0.0 100.0 0.0 - 0.0 0.0 0.0 0.0
SC 12.5 25.0 - 25.0 12.5 0.0 25.0 0.0
IC 34.7 - 4.2 0.0 4.2 11.1 45.8 0.0
TT - 47.2 22.2 0.0 0.0 0.0 22.2 8.3
start
TT IC SC FS CR SO SS ST
end
F
Week 27
High seizures number
ST 0.0 0.0 50.0 0.0 0.0 50.0 0.0 -
SS 25.0 12.5 37.5 0.0 0.0 25.0 - 0.0
SO 50.0 37.5 12.5 0.0 0.0 - 0.0 0.0
CR 0.0 0.0 50.0 0.0 - 0.0 50.0 0.0
FS 0.0 0.0 0.0 - 0.0 0.0 0.0 0.0
SC 16.7 66.7 - 0.0 0.0 0.0 16.7 0.0
IC 29.2 - 0.0 0.0 6.3 6.3 45.8 12.5
TT - 62.5 0.0 0.0 0.0 0.0 25.0 12.5
start
TT IC SC FS CR SO SS ST
end

Supplementary Table S14. Behavioral changes between sham and stimulated animals, validation cohort (Mean±SD).

A

**Open field test**	**Week 8**		
**Type:**	Sham	SE	p value
**latency to enter inner area of the arena [s]**	40.9±95.0	51.6±43.6	p > 0.05
**latency to enter the central area [s]**	180.3±342.1	282.0±336.7	p > 0.05
**speed [cm/s]**	5.6±2.3	7.2±1.6	p > 0.05

B

**Novel object exploration test**	**Week 8**		
**Type:**	Sham	SE	p value
**latency to enter the inner area of the arena [s]**	78.5±132.7	25.1±27.8	p > 0.05
**latency to approach the novel object [s]**	132.0±157.0	132.4±139.4	p > 0.05
**mobility [s]**	112.1±30.8	141.5±37.2	p > 0.05

C

**Elevated plus maze test**	**Week 8**		
**Type:**	Sham	SE	p value
**number of entries into the closed arms**	8.7±3.3	12.6±3.6	p > 0.05
**number of entries into the open arms**	31±4.3	25.4±9.3	p > 0.05
**speed [cm/s]**	4.4±0.4	5.5±1.4	p > 0.05

D

**Morris water maze test**	**Week 9**		
**Type:**	Sham	SE	p value
**swimming time over the platform [s]**	2.2±.1.1	2.0±1.4	p > 0.05
**speed [cm/s]**	30.6±1.6	31.4±4	p > 0.05
**time spent in the target quadrant, q4 [s]**	27.0±4.8	24.3±4.7	p > 0.05
**time spent in quadrant 1 [s]**	8.0±4.4	6.8±2.1	p > 0.05
**time spent in quadrant 2 [s]**	4.6±3.8	7.3±3.6	p > 0.05
**time spent in quadrant 3 [s]**	14.4±3.1	16.5±3.0	p > 0.05

Supplementary Figure S15. Swimming strategies in Morris water maze test between the sham and stimulated animals, validation cohort. RODA analysis (Mean±SEM).

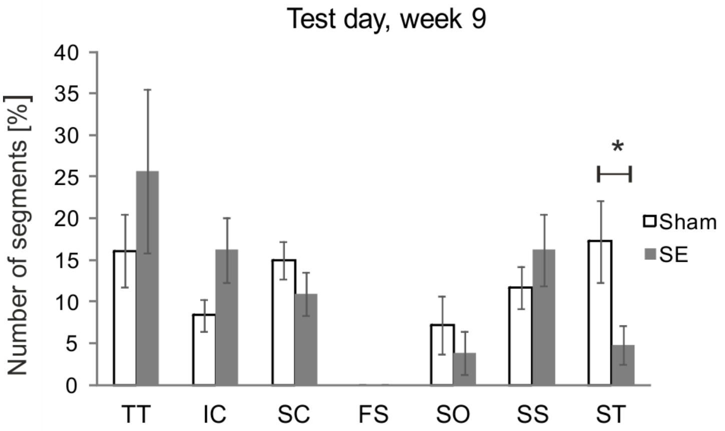

Supplement: Supplementary file 1 — Supplementary Information. [file 41598_2021_88088_MOESM1_ESM.pdf]
